# Structural, electronic, and gas adsorption properties of Ni_*n*_ (*n* = 1–20) atomic clusters

**DOI:** 10.1039/d6ra01586g

**Published:** 2026-04-23

**Authors:** Mohsen Doust Mohammadi, Nikolaos Patsalidis, Somnath Bhowmick, Vagelis A. Harmandaris, George Biskos

**Affiliations:** a Climate and Atmosphere Research Centre, The Cyprus Institute Nicosia 2121 Cyprus m.d.mohammadi@cyi.ac.cy s.bhowmick@cyi.ac.cy; b Computation-based Science and Technology Research Centre, The Cyprus Institute Nicosia 2121 Cyprus n.patsalidis@cyi.ac.cy; c Institute of Applied and Computational Mathematics (IACM), Foundation for Research and Technology Hellas, (FORTH), IACM/FORTH GR–71110 Heraklion Crete Greece v.harmandaris@cyi.ac.cy; d Department of Mathematics and Applied Mathematics, University of Crete Heraklion Crete GR–71409 Greece; e Faculty of Civil Engineering and Geosciences, Delft University of Technology Delft 2628 CN The Netherlands g.biskos@cyi.ac.cy g.biskos@tudelft.nl

## Abstract

Nickel clusters have drawn considerable interest because of their distinctive structural and electronic characteristics, which differ significantly from those of their bulk-material counterparts. In this work, we investigate the structures of Ni metal atomic clusters (Ni_*n*_, *n* = 1–20) in neutral charge state. We also explore how these clusters interact with a range of gases such as CO, CO_2_, CH_4_, NO, NO_2_, NH_3_, H_2_, H_2_O, N_2_, O_2_, and SO_2_, and compute the adsorption energies, in an effort to assess their potential exploitation in sensing materials. The geometries of the clusters are optimized, and the adsorption energies are calculated using the Density Functional Theory (DFT) method at the B3LYP-GD3BJ/LANl2DZ level of theory. Indicators, including cohesive energy, HOMO–LUMO energy gap, dissociation energy, and conceptual DFT analysis descriptors, show that the stability of these clusters increases with increasing size. Ni_19_ was found to be the most stable cluster among those that we studied, having the highest binding energy and a compact icosahedral geometry. As the size of the clusters increased, the cohesive energy increased, while the HOMO–LUMO gap decreased, indicating a transition from molecular to metallic behavior. The calculated adsorption energies revealed weak physisorption (0 to −1 eV) for CH_4_, H_2_, H_2_O, and N_2_, and strong chemisorption (−4 to −20 eV) for O_2_, NO, NO_2_, and SO_2_, with NO and NO_2_ binding most strongly on Ni_*n*_, for *n* = 16–19. Charge analysis indicates greater electron transfer and partial covalent bonding for the strongly adsorbed gases.

## Abstract 2

Geometries, electronic structure, and gas-adsorption properties of Ni_*n*_ atomic clusters with *n* = 1–20, using accurate density functional theory.

## Graphical abstract

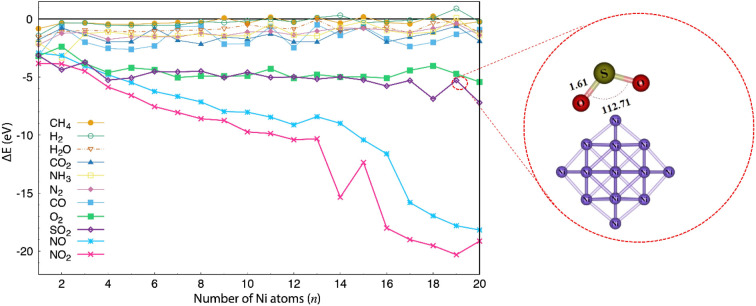

## 1 Introduction

Nickel (Ni) clusters represent an important class of materials because of their versatile structural and electronic properties. More specifically, they can function as tunable catalysts, where even the addition or removal of a single atom can significantly alter their electronic structure, thereby affecting their catalytic activity and selectivity in reactions such as hydrogenation and CO oxidation.^1,2^ In addition, their unique size-dependent geometries and stabilities provide opportunities to stabilize structural motifs, which are absent in bulk nickel, making them attractive building blocks for advanced nanomaterials.^3,4^ What is more, Ni clusters can exhibit strong interactions with gaseous species such as CO, NO, and H_2_, holding great potential in gas sensing and environmental remediation applications. Determining the structures of Ni clusters and understanding their reactivity with specific molecules are therefore crucial steps toward fully exploiting their potential use in different materials.

To understand how the properties of Ni atomic clusters vary with their size, we first need to determine their equilibrium structures. Theoretical investigations employing methods such as the Density Functional Theory (DFT) have shown that Ni clusters do not always follow the icosahedral growth pattern observed in other transition metals, but instead exhibit varied structural motifs such as square pyramids, tri-layered stacking patterns, and layered structures.^5^ Desmarais *et al.*^6^ performed first-principles electronic structure studies on Ni_7_ and Ni_8_ clusters, showing that the ground state of the former is a capped octahedron (binding energy 2.92 eV per atom), whereas that of the latter adopts a Jahn–Teller distorted *D*_2_ bisdisphenoid (3.01 eV per atom). Interestingly, both clusters also have several alternative structures that lie only 0.1–0.7 eV above their global minima, yet they all share the same magnetic moment (8.0 *µ*_B_), which is surprising given their different atomic arrangements.^7,8^ Complementing these findings, Chibani *et al.*^9^ conducted a systematic DFT study on small Ni_*n*_ clusters (*n* ≤ 15), showing that these occupy an intermediate state between isolated atoms and the bulk material, with unique geometrical configurations. Their results show that binding energies increase with cluster size, while magnetic moments oscillate, and that particularly stable clusters—such as Ni_2_, Ni_7_, Ni_9_, Ni_12_, and Ni_14_—prefer closed and highly symmetric structures. Despite the extensive body of work, questions remain regarding the detailed structural evolution, gas–cluster interactions, and size-dependent stability of Ni clusters under conditions relevant to applications in catalysis and sensing.

Computational methods that employ global optimization (GO) techniques provide effective tools to identify the most stable structures (ground state) among possible isomers.^10^ These methods efficiently explore the potential energy surface (PES) by moving between local minima, avoiding exhaustive point-by-point searches. GO approaches fall into two main categories: single-cluster methods (*e.g.*, basin hopping,^11^ simulated annealing^12^) and population-based methods, such as genetic algorithm,^13^ artificial bee colony (ABC),^14^ and particle swarming.^15^

We should note here that the large growth in structural isomers, followed by the increase in the size of the clusters, results in a vast expansion of local minima (LMs) within the PES. For instance, Zhang *et al.*^16^ identified two variants of Au_8_ as potential global minimum structures. Such examples demonstrate how structural complexity renders conventional local optimization methods, including gradient descent, conjugate gradient, steepest descent, and quasi-Newton approaches like BFGS, inadequate for comprehensive cluster structure determination, making GO techniques essential tools for uncovering stable structures that might otherwise remain hidden from conventional analysis.^17–21^

Expanding on the findings of previous studies,^22–24^ this work provides a comprehensive investigation by combining structural optimization and electronic structure characterization for a continuous series of neutral Ni_*n*_ (*n* = 1–20) clusters. Using the ABC algorithm,^16^ which are described in detail in the following sections, we provide a systematic analysis of the Ni_*n*_ cluster interaction with various atmospheric gases such as CO, CO_2_, CH_4_, NO, NO_2_, NH_3_, H_2_, H_2_O, N_2_, O_2_, and SO_2_. The nature of interaction between clusters of different sizes with the adsorbate gases is also evaluated using various wave function analysis methods, including Natural Population Analysis (NPA) and Quantum Theory of Atoms in Molecules (QTAIM).

## 2 Computational details

All calculations were carried out using the B3LYP^25^ DFT method with Grimme's D3 dispersion correction,^26^ including Becke–Johnson damping (GD3BJ). The LANL2DZ^27^ basis set was employed for all atoms, providing effective core potentials for heavier elements while maintaining computational efficiency. The geometries of the clusters were optimized without any symmetry constraints using the BFGS algorithm with tight convergence criteria (energy threshold: 10^−8^ Hartree, gradient threshold: 10^−6^ Hartree/Bohr, displacement threshold: 10^−6^ Bohr). Following the geometry optimization, vibrational frequency calculations were performed at the same level of theory to confirm the nature of the stationary points (absence of imaginary frequencies for minima) and to obtain zero-point energy and thermal corrections.

The B3LYP-GD3BJ functional was selected over other functionals, such as the widely used ωB97XD, because it demonstrates superior convergence, particularly for larger Ni clusters, where calculations with the ωB97XD functional frequently encounter SCF convergence difficulties or fail entirely. Similarly, we used the LANL2DZ basis set as it provides an optimal balance between computational efficiency and accuracy for transition metal systems. LANL2DZ has been extensively benchmarked for Ni-containing systems,^28^ demonstrating reliable performance in predicting structural and energetic properties.^29,30^ For all the electronic structure calculations, we employed the Gaussian 16 software package.^31^ We considered only the lowest spin states, where closed-shell systems were treated as singlet spin states (*S* = 0), whereas open-shell systems were treated as doublet spin states (*S* = 1/2).

We employed conceptual DFT (CDFT) to investigate the chemical reactivity and stability of the clusters by calculating key global electronic descriptors, including electronegativity, the highest occupied molecular orbital (HOMO) – lowest unoccupied molecular orbital (LUMO) energy gap, chemical hardness (*η*), chemical potential (*µ*), and electrophilicity index (*ω*).^32,33^ Details of the conceptual DFT descriptors used in this study are provided in the literature.^34–36^

The cohesive energy per atom, which is a critical measure of the stability of atomic clusters, was estimated as:1
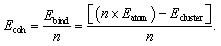
Here, *E*_bind_ is the total binding energy of the cluster, *n* is the number of Ni atoms in the cluster, *E*_atom_ denotes the energy of a single free Ni atom, and *E*_cluster_ is the total energy of the cluster. By comparing cohesive energies, we can assess the relative stability of the clusters.^37,38^

The dissociation of Ni_*n*_ clusters with the selective formation of Ni_*n*−1_ or Ni_*n*−2_ clusters is another approach to evaluate the relative stability of the clusters. In general, from the total energy of the clusters, the dissociation energy (*D*_e_) can be obtained by considering the following reactions:

2 Ni_*n*_ + *D*_e,1_ → Ni_*n*−1_ + Ni

3 Ni_*n*_ + *D*_e,2_ → Ni_*n*−2_ + Ni_2_

Here, *D*_e,1_ represents the energy required for the dissociation of a Ni_*n*_ into a Ni_*n*−1_ cluster by removing one Ni atom, and *D*_e,2_ represents the energy required for the dissociation of a Ni_*n*_ into a Ni_*n*−2_ by removing a Ni_2_ dimer. The higher the values of *D*_e,1_ and *D*_e,2_, the higher the stability of the corresponding cluster against the specific dissociation channels.

After determining the structure and stability of the clusters, we investigated how these interact with various gaseous species. More specifically, we determined the energy of adsorption of a gas molecule onto the cluster (*i.e.*, the net energy released/needed when a gas molecule adsorbs on the surface of any metal cluster) as follows:

4 *E*_ads_ = *E*_gas@cluster_ − *E*_cluster_ − *E*_gas_ + Δ*E*_ZPE_

Here, *E*_gas@cluster_ denotes the total electronic energy of the most stable gas molecule, adsorbed on the cluster, *E*_cluster,_ and *E*_gas_ refers to the total electronic energy of the isolated cluster and the gas molecule, respectively, whereas Δ*E*_ZPE_ is the energy correction accounting for the zero-point vibrational energy (ZPE) contributions. We should note that the notation gas@cluster denotes a gas molecule adsorbed on the surface of the Ni cluster, where the gas molecule is the adsorbate and the cluster is the substrate.

The ABCluster software^14,39^ that implements the global optimization algorithm of the artificial bee colony,^40^ was used to systematically determine the minimum energy structures of the Ni_*n*_ clusters. As a first step, we generated 300 000 pure Ni cluster structures and performed energy minimization calculations using the Gupta potential,^41^ which is a well-established model for metallic systems. The specific parameter values of the Gupta potential we used were adopted from previously published literature,^42^ after validation against experimental and high-level computational data; the values are provided in Table S1 of the SI. Among all of the structural isomers, those with similar symmetries and energy values were screened and grouped together, as they are likely to have comparable geometries. From each group, one cluster was selected to represent the symmetrically distinct structures. This approach helped eliminate duplicates based on energy similarity. The selected representative clusters were then introduced to the next level of geometry optimization simulations for a more detailed semi-empirical screening using the PM6 (ref. 43) method within the xtb software.^44^ Finally, a few of the lowest energy optimized clusters obtained at the previous step, whose number varied depending on cluster size, were optimized at the DFT level (with B3LYP-GD3BJ/LANL2DZ) using the Gaussian 16 software. After optimization, we carried out vibrational harmonic frequency calculations at the DFT level in order to verify local minima and to determine ZPE values. A similar approach was employed to find the most stable gas–cluster structures and their electronic energies.

## 3 Discussion and results

### 3.1 Structure of the Ni clusters

**Fig. 1:**
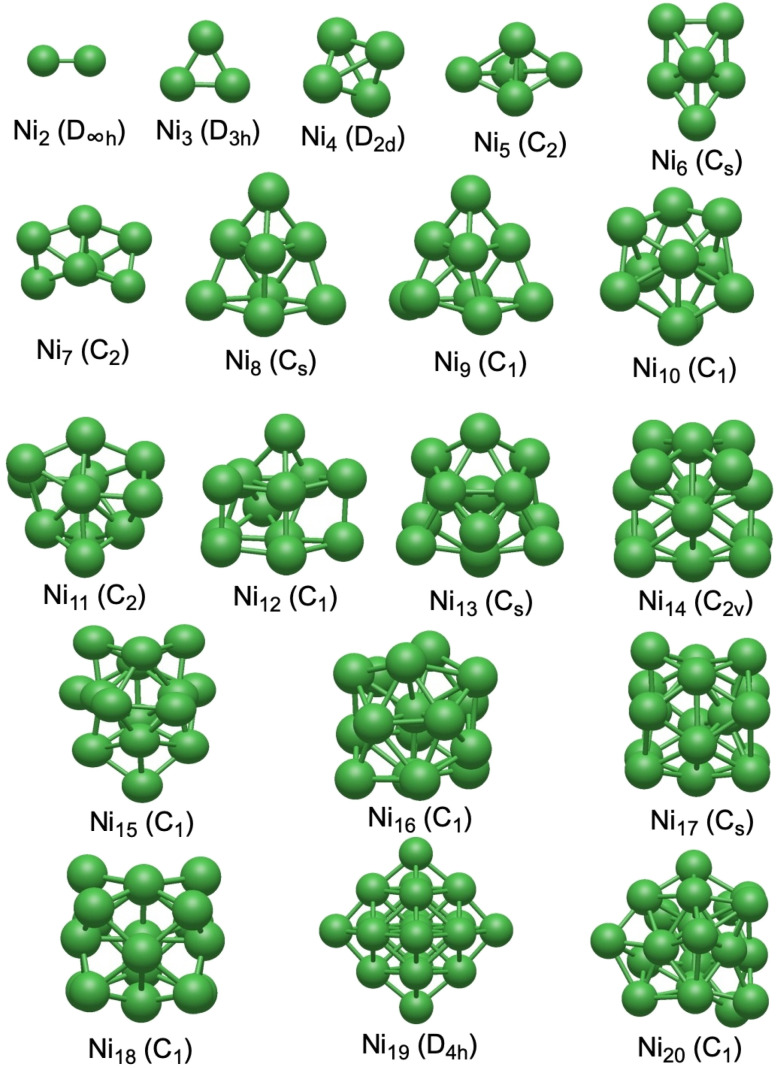
Illustrations of the energetically most stable isomers of Ni_*n*_ atomic clusters (*n* = 2–20), as determined by the geometric optimization at the B3LYP-GD3BJ/LANl2DZ level. Point group symmetries are provided in parentheses.

Fig. 1 illustrates the most stable structures for neutral Ni clusters containing 2 to 20 atoms, obtained using the B3LYP-GD3BJ/LANL2DZ method. All optimized Cartesian coordinates for these stable structures are provided in Table S2 of the SI. Each structure represents the global minimum energy isomer identified through a comprehensive search, as described in the methodology section. The structures of different isomers for specific cluster sizes are labelled with suffixes “_01”, “_02”, *etc.*, and are provided in Fig. S1 to S14 of the SI. Additionally, Table S3 in the SI provides the zeropoint energies and ZPE-corrected electronic energies (EE) of various optimized isomers of Ni clusters (*n* = 1–20).

The ZPE rises monotonically with increasing *n*, exhibiting a modest variation across the isomers for a given size. For example, the ZPE for Ni_8_ ranges from approximately 0.20 to 0.27 eV, while for Ni_20_, it ranges from 0.60 to 0.81 eV. This increase reflects the growing number of vibrational modes and their softening as the cluster size increases. For a fixed number of atoms, the ZPE ranges from a few hundredths (10^−2^) to a few tenths (10^−1^) of an eV, which is significant for differentiating between nearly degenerate structures.

For *n* ≥ 4, the low-lying region of the potential energy surface is relatively shallow, with several isomers found within 0.1 eV of the global minimum isomer, occasionally resulting in near-degeneracies at the current level of theory. For example, for Ni_4_, two isomers (Ni_4__01 and Ni_4__03) have the same electronic energy, while Ni_4__02 is higher by 0.168 eV. For Ni_10_, the energy difference among the lowest energy isomers is minimal: *e.g.*, Ni_10__02 (−46 171.804 eV) and Ni_10__05 (−46 171.803 eV) have energies that can be considered the same within numerical uncertainties. For Ni_12_, the global minimum isomer (Ni_12__12, −55 407.721 eV) has an energy that is only 0.143 eV lower compared to the next lowest energy isomer (Ni_12__01). For larger clusters, multiple minima reemerge; for instance, Ni_17__10 and Ni_17__16 both have energies of −78 497.77 eV. It should be noted that the overall energy spread among isomers within a specific size can reach approximately 1 to 3 eV. For Ni_17_, the global minimum has an energy of −78 497.772 eV compared to the highest energy isomer at −78 496.070 eV, and for Ni_20_, the global minimum is at −92 351.575 eV, while the highest energy isomer is at −92 348.512 eV. In the rest of the paper, the discussion is restricted to the global minimum structure of each Ni_*n*_.

Comparison of the calculated average bond lengths (see Table S4 in the SI) demonstrates good agreement with previous studies that examined only limited cluster sizes. Smaller clusters show considerable bond contraction, with Ni_2_ exhibiting the shortest bond length at 2.09 Å, which is comparable to the value of 2.15 Å reported by Pinegar *et al.*^45^ As the cluster size increases, the bond lengths gradually increase: for example, Ni_3_ has an average bond length of 2.22 Å, matching the result provided by Erkoç *et al.*,^46^ whereas Ni_4_, with an average bond length of 2.35 Å, falls within the reported range of 2.23–2.41 Å.^47^

For medium-sized clusters, our results are consistent with previous computational studies, especially those by Chibani *et al.*^9^ Specifically, we found that the bond lengths estimated here are in good agreement with those reported by Chibani *et al.*: *e.g.*, Ni_5_ (2.36 Å *vs.* 2.32 Å), Ni_6_ (2.36 Å *vs.* 2.35 Å), and Ni_13_ (2.45 Å *vs.* 2.44 Å). The largest deviations were observed for Ni_7_ (2.33 Å) compared to the values reported by Nayak *et al.* (2.38–2.43 Å),^48^ and for Ni_9_ (2.407 Å) compared to the broader range estimated by Chibani *et al.* (2.33–2.57 Å).^9^

For larger clusters (Ni_14_–Ni_20_), bond lengths stabilize around 2.41–2.47 Å, remaining close to the bulk value of the Ni–Ni bond length, which is 2.49 Å.^49^ It is important to note that these comparisons are made between computational studies that employed different functionals and basis sets, so subtle discrepancies are to be expected. To the best of our knowledge, experimental bond length data for these cluster sizes are not available in the literature.

The increase in average Ni–Ni bond length with cluster size arises from the gradual increase in coordination number and the transition toward more delocalized metallic bonding. In small clusters, low coordination leads to higher bond order per bond and bond contraction, whereas in larger clusters, bonding interactions are distributed over more neighbours, reducing bond order per bond and increasing bond length toward the bulk value (∼2.49 Å).

### 3.2 Stability analysis

**Fig. 2:**
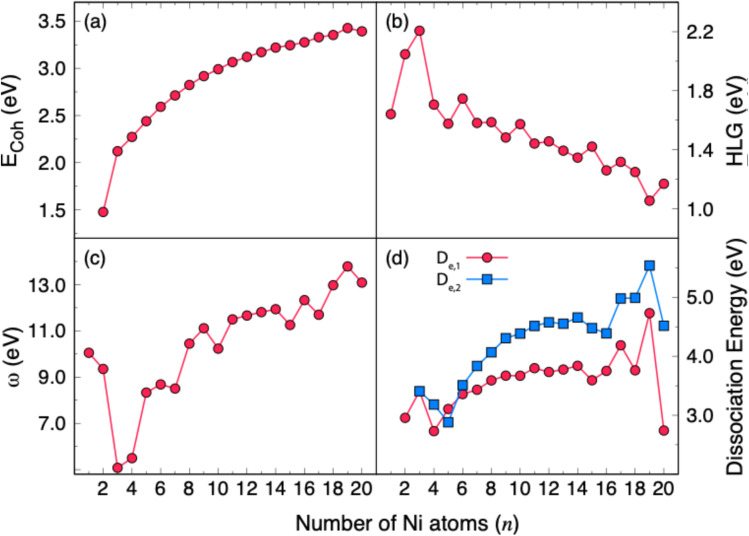
Evolution of (a) the cohesive energy per atom (*E*_coh_), (b) the HOMO–LUMO energy gap (HLG), (c) the electrophilicity index (*ω*), and (d) the dissociation energy (*D*_e_) for the loss of Ni_1_ and Ni_2_, as a function of the number of atoms (*n*) in the Ni clusters. All calculations were performed at the B3LYP-GD3BJ/LANL2DZ level on the global minimum structure of Ni_*n*_ clusters, where *n* = 1–20.

The cohesive energy per atom (*E*_coh_) of the Ni_*n*_ clusters (for *n* = 1–20), as shown in Fig. 2a, demonstrates a clear size dependence, reflecting a progressive stabilization and transition toward bulk-like metallic behavior with increasing size. For small clusters (up to 3–4 atoms), these values increase rapidly, with the most significant *E*_coh_ jump occurring between Ni_2_ and Ni_3_. As the size of the clusters grows, the cohesive energy begins to plateau, indicating that each additional atom contributes less to overall stability. However, there are notable deviations in the *E*_coh_ trend. A local maximum is predicted for the Ni_19_ cluster in our calculations, indicating enhanced stability of Ni_19_ relative to its immediate neighbors. Ni_20_ displays a slight decrease in cohesive energy (3.39 eV) relative to Ni_19_ (3.42 eV), indicating that within the set of optimized structures considered here, Ni_19_ is more strongly bound on a per-atom basis than Ni_20_, which could be linked to its highly symmetric structure.

When comparing the cohesive energies determined in this study to those reported by Chibani *et al.*,^9^ we observe a consistent systematic deviation. Although both datasets follow a similar increasing trend with cluster size (see Table S5), our results are slightly lower in magnitude, with Ni_3_ being the only exception. The deviations of the cohesive energies from those reported by Chibani *et al.*^9^ range from 1.64 to 7.59%. This difference may be attributed to methodological factors, such as variations in the implementation of the exchange–correlation functional, convergence parameters, or numerical settings. Nonetheless, the close agreement between the two datasets suggests that the essential physical trends in Ni_*n*_ clusters are reliably reproduced.

The HOMO–LUMO energy gap (HLG) for the Ni_*n*_ clusters (*n* = 1–20), summarized in Fig. 2b and Table S6, also reveals significant size-dependent variations. The calculated HLGs range from a maximum of 2.21 eV for Ni_3_ to a minimum of 1.05 eV for Ni_19_. The notably large gap for Ni_3_ indicates enhanced electronic stability and chemical inertness. In contrast, as the clusters increase in size, the HLG generally decreases. For example, clusters larger than Ni_13_ exhibit HLGs below 1.40 eV, suggesting enhanced reactivity. However, this trend is not strictly monotonic. For example, intermediate-sized clusters such as Ni_4_, Ni_6_, and Ni_10_ show larger HLGs than their immediate neighbors. Similarly, Ni_14_ and Ni_16_ have slightly smaller HLG values than their neighbors. Overall, these results indicate that smaller clusters (*n* ≤ 8) are electronically more stable and chemically less reactive due to their relatively larger HLGs. In contrast, larger clusters (*n* ≥ 13) tend to the properties of bulk nickel, as indicated by the narrower HLGs and higher reactivities.

The electrophilicity index (*ω*) of the Ni_*n*_ clusters is presented in Fig. 2c. Detailed methodology for the calculation of *ω* is provided in the SI. The values range from 5.08 eV for Ni_3_ to 13.78 eV for Ni_19_, indicating a progressive increase in electrophilic character as the clusters grow in size. The notably low value for Ni_3_ is directly related to its large HLG (2.21 eV), indicating a reduced tendency to accept electrons. In contrast, larger clusters, particularly those larger than Ni_10_, exhibit significantly higher *ω* values (approximately 11 eV or higher). Intermediate-sized clusters, such as Ni_5_, Ni_6_, and Ni_7_, display *ω* values in the range 8–9 eV, lying between these two extremes. In the *n* = 16–19 range, *ω* increases markedly and reaches its maximum at Ni_19_ (13.78 eV), which also corresponds to the smallest HLG (1.05 eV).

The dissociation energies for the first (*D*_e,1_) and second (*D*_e,2_) atom-removal steps of Ni_*n*_ clusters are shown in Fig. 2d. In general, both *D*_e,1_ and *D*_e,2_ increase with cluster size over the range considered, with the increase of *D*_e,2_ being more pronounced than that of *D*_e,1_. This suggests that once a cluster exceeds *ca.* 8–10 Ni atoms, removing two atoms at the same time becomes significantly more difficult. For very small clusters such as Ni_3_–Ni_5_, the second dissociation energy is only about 5–15% higher than the first. In contrast, for larger clusters (*n* ≥ 10), *D*_e,2_ exceeds *D*_e,1_ by 20–30%, and for Ni_19_, the second dissociation energy is nearly 30% higher. Interestingly, Ni_19_ exhibits the highest values of both *D*_e,1_ and *D*_e,2_, whereas the next larger cluster, Ni_20_, has one of the lowest *D*_e,1_ values among the clusters considered.

### 3.3 Adsorption energy trends

**Fig. 3:**
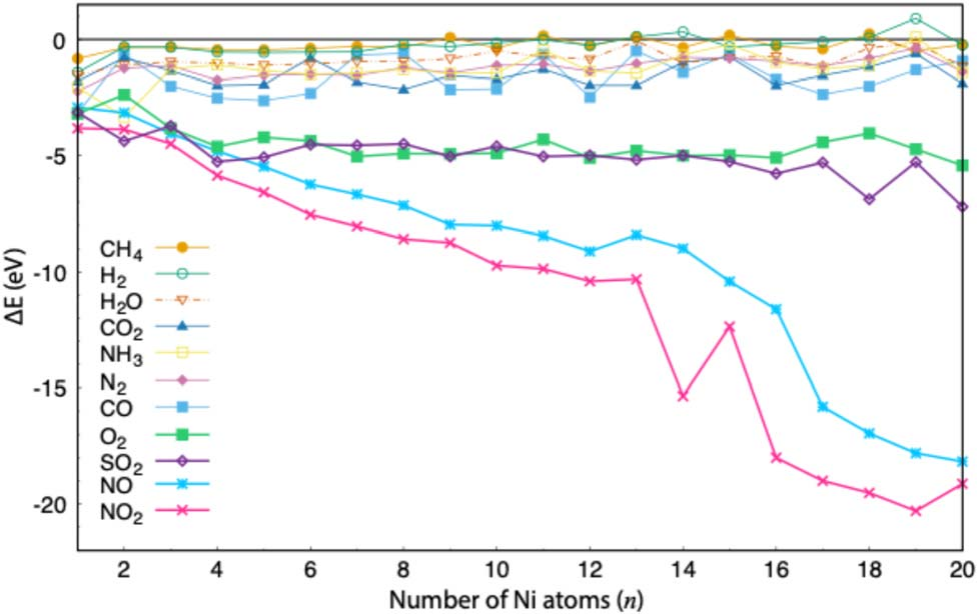
Adsorption energies (*E*_ads_) of the CH_4_, CO, CO_2_, H_2_, H_2_O, N_2_, NH_3_, NO, NO_2_, O_2_, and SO_2_ gases on the Ni_*n*_ (*n* = 1–20) clusters. Calculations have been performed at the B3LYP-GD3BJ/LANl2DZ level. Numerical values of *E*_ads_ can be found in Table S7 in the SI.

The adsorption properties of different gases on Ni clusters (Ni_1_–Ni_20_) reveal two distinct patterns. As illustrated in Fig. 3 and shown in Table S7, gases such as CH_4_, H_2_, H_2_O, and N_2_ exhibit weak adsorption energies, with some *E*_ads_ values close to zero or slightly positive. Such small magnitudes of *E*_ads_ are characteristic of physisorption dominated by dispersion forces, with negligible orbital overlap or charge transfer. As a result, these molecules remain largely inactivated in the Ni clusters.

In contrast, CO, CO_2_, and NH_3_ show intermediate adsorption energies indicative of mixed physisorption–chemisorption character. CO, in particular, engages in σ-donation from the 5σ orbital and π back-donation into the 2π* orbital, while NH_3_ interacts primarily through a lone-pair donation from the N-centered sp^3^ hybrid orbital. Similarly, O_2_ and SO_2_ exhibit consistently larger adsorption energies and clear chemisorption signatures throughout the size range. The corresponding *E*_ads_ values, typically in the range of *ca.* −4 to −7 eV, align well with the requirements for catalytic activation and bond cleavage, further highlighting the potential of Ni clusters as active sites for oxidation processes.^50^

The strongest adsorption occurs with NO and NO_2_, which bind exceptionally well to Ni clusters as the cluster size increases. We found that the *E*_ads_ values for NO become more exothermic from about −2.9 eV on Ni_1_ to roughly −12 to −18 eV on Ni_16_–Ni_20_, while NO_2_ reaches values as high as −20.3 eV on Ni_19_. These adsorption energies suggest strong chemisorption with possible multiple bonding interactions. Although this implies a high reactivity of Ni_*n*_ clusters toward these two gases, it also suggests a strong tendency for irreversible site blocking and catalyst poisoning.

The strong interactions of O_2_, NO, and NO_2_ with Ni clusters, in comparison to closed-shell gases, can be attributed to their open-shell (paramagnetic) electronic structures. Triplet O_2_, doublet NO, and doublet NO_2_ each contain one or more unpaired electrons, leading to enhanced spin density at the gas–cluster interface and enabling strong spin-dependent exchange interactions with Ni d orbitals. This facilitates more effective orbital hybridization and charge transfer, which in turn increases the strength of chemisorption. In contrast, closed-shell molecules do not benefit from this additional spin-exchange stabilization and generally exhibit weaker adsorption. These conclusions are supported by previous studies demonstrating that NO adsorption on NiO alters local magnetic moments and spin polarization, directly impacting binding energies, and that open-shell adsorbates induce stronger spin-dependent perturbations than closed-shell molecules.

Among the closed-shell gases considered here, SO_2_ exhibits by far the strongest adsorption in Ni_*n*_ clusters. This can be attributed to the combination of the electronic structure and the molecular geometry. The relatively high electronegativity of oxygen (and the moderately high electronegativity of sulfur relative to Ni) promotes substantial charge polarization and charge transfer upon interaction with the cluster, enhancing the electrostatic and covalent nature of bonding. Previous studies have emphasized the importance of electronegativity, charge transfer, and orbital interactions in determining adsorption energies of gases on Ni and other transition metal clusters (Araujo *et al.*^51^). Furthermore, the compact and bent geometry of SO_2_ minimizes steric hindrance, allowing multiple atoms (the sulfur and both oxygens) to interact closely with the cluster, thus maximizing contact area, potential bonding sites, and overlap between the SO_2_ frontier orbitals and the Ni d orbitals.

The comparatively weaker adsorption of CH_4_, H_2_, H_2_O, and N_2_, and the moderate binding of CO, CO_2_, and NH_3_, relative to NO, NO_2_, O_2_, and SO_2_, can be explained by several factors related to their molecular properties, electronic configurations, and steric effects. First, many of these molecules (CH_4_, H_2_, and N_2_) are nonpolar or possess very low polarity, which limits electrostatic interactions with the Ni clusters and favors dispersion-dominated physisorption. Secondly, while some molecules, such as CO and H_2_O, do have permanent dipole moments, their orientations may not be optimal for strong interactions with the Ni surface, or the molecular geometry may partially neutralize their dipole moments. Steric factors can also play a role. For example, the pyramidal geometry of NH_3_ can limit its ability to approach specific Ni sites as smaller linear molecules (*e.g.*, O_2_ or NO), further reducing the maximum achievable binding energy.

### 3.4 Natural population analysis

Three gases, *viz.*, CO_2_, SO_2_, and NO_2_, were selected for NPA (see SI for a detailed methodology). These gases were chosen because they exhibit distinct electronic and structural features—CO_2_ is linear and nonpolar, SO_2_ is a closed-shell but chemically reactive species, and NO_2_ is an open-shell radical with an unpaired electron. They also span a wide range of adsorption energies on Ni clusters, with CO_2_ showing relatively low, SO_2_ moderate, and NO_2_ high adsorption energies, as described in the previous section. Among all the investigated clusters, Ni_19_ was identified as the most stable and was therefore used as a system to probe general charge-transfer properties across different adsorbates. In addition, Ni_4_ was examined specifically for the NO_2_ system to enable a direct comparison between a small and a larger cluster. In this case, NO_2_ adsorption on Ni_4_ is significantly weaker than on Ni_19_, providing a useful contrast in adsorption energies.

The NPA results (Table 1) reveal a clear correlation between net charge transfer (Δ*q*) and adsorption energy. The total charge transferred from the Ni_19_ cluster to the adsorbed molecule increases in the order CO_2_ (Δ*q* = −0.66) < SO_2_ (Δ*q* = −0.68) < NO_2_ (Δ*q* = −1.06), which is consistent with the calculated trend in adsorption energy. For CO_2_, only moderate changes were observed in the natural charges and electron configurations. The positive charge on the carbon atom decreased from +1.02 in the gas phase to +0.65 after adsorption, while both oxygen atoms became slightly more negative (from −0.51 to −0.65 and −0.66). These changes were accompanied by minor increases in the 2p orbital occupations, indicating limited back-donation and a relatively weak interaction with the Ni_19_ cluster.

**Table 1:** Natural electron configurations and atomic charges of CO_2_, NO_2_, and SO_2_ gas molecules, and when those are adsorbed on Ni_4_ and Ni_19_ clusters. Calculations have been performed at the B3LYP-GD3BJ/LANl2DZ level. Δ*q* is the total electronic charge of the gas molecule after adsorption

System	Atom	Natural electron configuration	Charge	Δ*q*
CO_2_	C	[Core]2s(0.69)2p(2.28)3p(0.02)	1.01612	
	O	[Core]2s(1.77)2p(4.73)3p(0.01)	−0.50806	
	O	[Core]2s(1.77)2p(4.73)3p(0.01)	−0.50806	
CO_2_@Ni_19_	C	[Core]2s(0.93)2p(2.38)3s(0.01)3p(0.03)	0.65002	−0.66
	O	[Core]2s(1.72)2p(4.93)3p(0.01)	−0.65491	
	O	[Core]2s(1.72)2p(4.93)3p(0.01)	−0.66013	
SO_2_	S	[Core]3s(1.74)3p(2.91)4s(0.01)4p(0.02)	1.32459	
	O	[Core]2s(1.91)2p(4.74)	−0.66229	
	O	[Core]2s(1.91)2p(4.74)	−0.66229	
SO_2_@Ni_19_	S	[Core]3s(1.68)3p(3.38)4p(0.01)	0.92703	−0.68
	O	[Core]2s(1.85)2p(4.93)3p(0.01)	−0.78086	
	O	[Core]2s(1.81)2p(5.00)3p(0.01)	−0.82191	
NO_2_	N	[Core]2s(1.31)2p(3.18)3s(0.02)3p(0.02)	0.47925	
	O	[Core]2s(1.80)2p(4.42)3p(0.01)	−0.23962	
	O	[Core]2s(1.80)2p(4.42)3p(0.01)	−0.23962	
NO_2_@Ni_19_	N	[Core]2s(1.56)2p(3.26)3p(0.02)	0.15356	−1.06
	O	[Core]2s(1.77)2p(4.89)3p(0.01)	−0.67301	
	O	[Core]2s(1.77)2p(4.76)3p(0.01)	−0.53799	
NO_2_@Ni_4_	N	[Core]2s(1.54)2p(3.01)3s(0.01)3p(0.02)	0.41376	−0.63
	O	[Core]2s(1.77)2p(4.73)3p(0.01)	−0.51129	
	O	[Core]2s(1.77)2p(4.74)3p(0.01)	−0.52888	

In contrast, SO_2_ and NO_2_ exhibit more pronounced electronic interactions with the cluster. For SO_2_, the positive charge of the sulfur atoms decreases from +1.32 to +0.93 upon adsorption, while the oxygen atoms becomes significantly more negative (−0.78 and −0.82), with notable increases in the 3p (S) and 2p (O) orbital populations, suggesting stronger bonding interactions with the Ni_19_ cluster. The largest charge redistribution is determined for NO_2_, where the nitrogen atom becomes substantially less positive (from +0.48 to +0.15), and the oxygen atoms gain greater negative charge (−0.67 and −0.54), accompanied by increased 2p occupations. These changes are associated with the largest Δ*q* among the three gases.

Furthermore, when comparing the adsorption of NO_2_ on Ni_4_ and Ni_19_ clusters, the total charge transfer was significantly higher for Ni_19_ (Δ*q* = −1.06 *vs.* −0.63), with the nitrogen atom retaining a much higher positive charge (+0.41) on Ni_4_ than on Ni_19_ (+0.15). Taken together, these results demonstrate that stronger adsorption correlates directly with greater electron transfer from the cluster. This confirms that charge transfer plays a pivotal role in the determination of adsorption energy, with NO_2_ exhibiting the strongest interaction, followed by SO_2_ and CO_2_.

### 3.5 QTAIM analysis

**Fig. 4:**
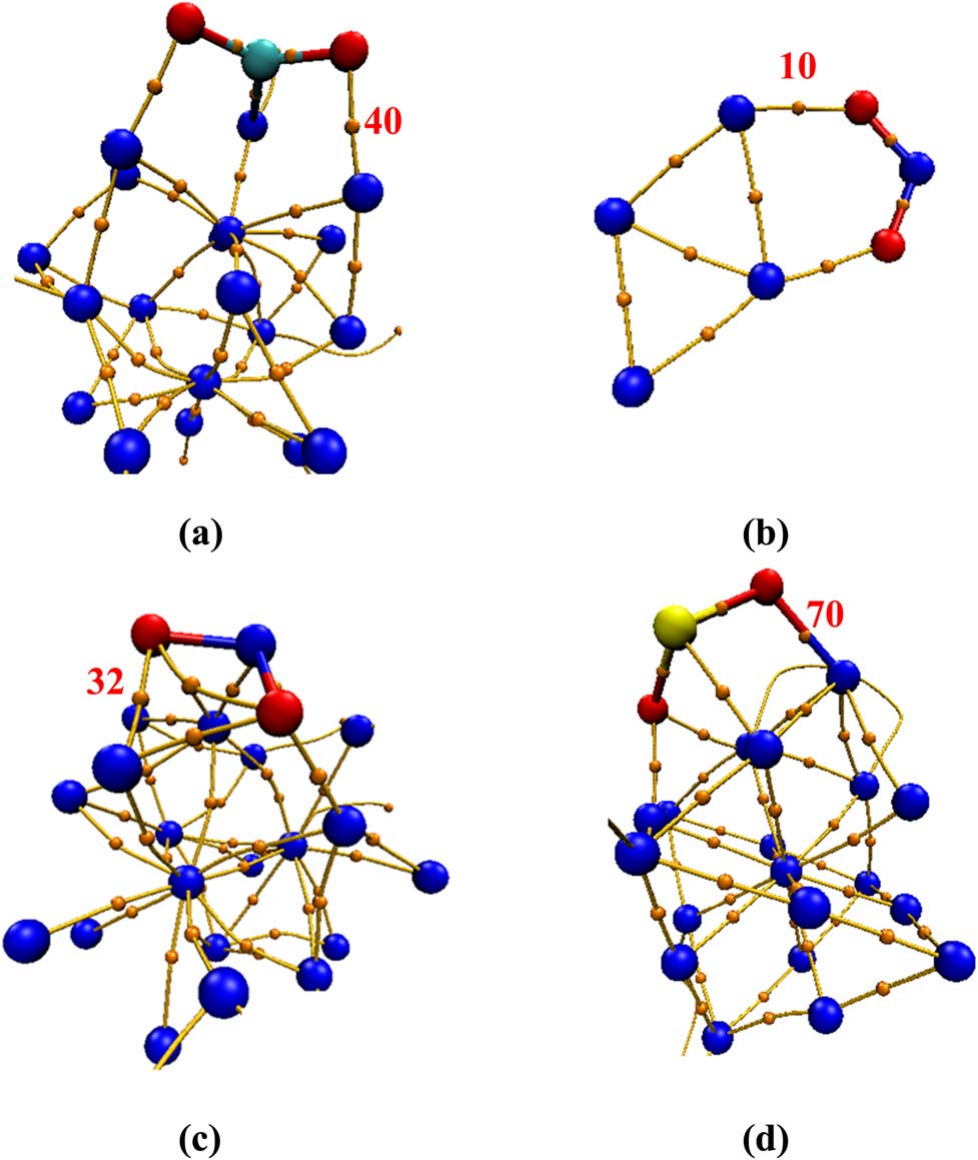
Illustrations showing structures of gas@cluster systems with their critical bond points, denoted by small orange spheres, and their numbers for (a) CO_2_@Ni_19_; (b) NO_2_@Ni_4_; (c) NO_2_@Ni_19_; and (d) SO_2_@Ni_19_. Calculations have been performed at the B3LYP-GD3BJ/LANl2DZ level of theory.

Fig. 4 provides example illustrations of gas–cluster systems, including the locations of critical bond points (BCPs) identified during the topological analysis of electron density for the CO_2_, NO_2_, and SO_2_ adsorbed on Ni clusters. Table 2 summarizes the corresponding electron-density descriptors at these BCPs. The BCPs highlighted in Fig. 4 correspond to the dominant gas–cluster contacts, *i.e.*, regions where electron density is concentrated between interacting gas and cluster atoms, and thus directly characterize the strength and nature of the bonding between the adsorbed molecules and the Ni_4_ or Ni_19_ clusters.

**Table 2:** Electron density parameters at critical bond points for CO_2_, NO_2_, and SO_2_ adsorption on Ni_4_ and Ni_19_ clusters. Parameters include bond critical point (BCP) number, electron density *ρ*(**r**), kinetic energy density *G*(**r**), potential energy density *V*(**r**), Laplacian of electron density ∇^2^*ρ*(**r**), Electron Localization Function (ELF), Localized Orbital Locator (LOL), and energy density ratio *G*(**r**)/*V*(**r**). All values are reported in atomic units (a.u.). Calculations have been performed at the B3LYP-GD3BJ/LANl2DZ level

System	BCP number	*ρ*(**r**)	*G*(**r**)	*V*(**r**)	∇^2^*ρ*(**r**)	ELF	LOL	*G*(**r**)/*V*(**r**)
CO_2_@Ni_19_	40	0.1190	0.0705	−0.1058	0.1410	0.5785	0.5395	−0.6665
NO_2_@Ni_4_	10	0.0328	0.0320	−0.0359	0.1129	0.0907	0.2357	−0.8932
NO_2_@Ni_19_	32	0.0662	0.1921	−0.1956	0.7543	0.0309	0.1448	−0.9821
SO_2_@Ni_19_	70	0.0726	0.2210	−0.2291	0.8513	0.0262	0.1409	−0.9644

As shown in the results provided in Table 2, the SO_2_@Ni_19_ system has the highest electron density with a *ρ*(**r**) value of 0.0726 a.u. and a large positive Laplacian of electron density; *i.e.*, ∇^2^*ρ*(**r**) of 0.8513 a.u. This is indicative of a mixed covalent-ionic interaction with substantial charge accumulation between the interacting atoms. The NO_2_@Ni_19_ system also shows a sizeable electron density, *ρ*(**r**) = 0.0662 a.u., and a similarly large ∇^2^*ρ*(**r**) of 0.7543 a.u., consistent with a partially covalent but somewhat less localized bond compared to SO_2_. In contrast, NO_2_@Ni_4_ displays a much smaller *ρ*(**r**) value of 0.0328 a.u., reflecting weaker bonding, whereas CO_2_@Ni_19_ has a moderately high *ρ*(**r**) with a value of 0.1190 a.u., indicating a stable yet relatively polar interaction.

Further insights into the bonding nature can be gained from the kinetic energy density (*G*(**r**)), potential energy density (*V*(**r**)), and their ratio *G*(**r**)/*V*(**r**), supported by the Electron Localization Function (ELF) and Localized Orbital Locator (LOL) values. Ratios of *G*(**r**)/*V*(**r**) close to −1 typically indicate covalent interactions, while values approaching zero correspond to electrostatic or weak interactions. As shown in Table 2, the SO_2_@Ni_19_ system (*G*(**r**)/*V*(**r**) = −0.9644) and NO_2_@Ni_19_ (*G*(**r**)/*V*(**r**) = −0.9821) both exhibit ratios close to −1, confirming the strong covalent character. Conversely, CO_2_@Ni_19_ (*G*(**r**)/*V*(**r**) = −0.6665) and NO_2_@Ni_4_ (*G*(**r**)/*V*(**r**) = −0.8932) display weaker covalent contributions.

The ELF and LOL values further corroborate these observations. SO_2_@Ni_19_ has low ELF (0.0262) and LOL (0.1409) values, suggesting electron delocalization due to strong bonding interactions. In contrast, CO_2_@Ni_19_, with higher ELF (0.5785) and LOL (0.5395), indicates localized electron regions associated with moderate bonding. Collectively, these QTAIM descriptors reveal that SO_2_ forms the most covalent and tightly bound interaction with Ni_19_, followed by NO_2_@Ni_19_ and CO_2_@Ni_19_, while NO_2_@Ni_4_ exhibits the weakest bonding.

## 4 Conclusions

We have investigated the structural, electronic, and gas adsorption properties of nickel clusters (Ni_*n*_; *n* = 1–20) through comprehensive DFT calculations and determined fundamental size-dependent properties that govern their stability and capability to adsorb gas molecules. Our analysis shows that the clusters exhibit distinct evolutionary patterns as their size increases. Specifically, the cohesive energy progressively increases while the HOMO–LUMO gap decreases, exhibiting a clear transition from molecular to bulk metallic behavior. This transition is accompanied by an enhancement in the electrophilicity in larger clusters, which indicates superior electron-accepting capabilities.

When interacting with gaseous species, the Ni clusters demonstrate a distinct behavior. Molecules, such as CH_4_, H_2_, H_2_O, and N_2_, show weak physisorption with adsorption energies (*E*_ads_) ranging from approximately 0 to −1 eV. In contrast, reactive species including O_2_, NO, NO_2_, and SO_2_ exhibit strong chemisorption, with *E*_ads_ values ranging between −4 to −20 eV. Notably, NO and NO_2_ bind most strongly, with *E*_ads_ reaching up to −20.3 eV on Ni_16_–Ni_19_, suggesting high reactivity. O_2_ and SO_2_ have moderate adsorption energies (−4 to −7 eV), suggesting less favourable oxidation by these species. NPA analysis shows that stronger adsorption arises from greater electron transfer from the Ni cluster to the gas molecule, while QTAIM results confirm that these stronger interactions have a stronger covalent character, especially for larger clusters.

Overall, our calculations reveal clear structure–property relationships and size-dependent properties of the Ni_*n*_ clusters. Among the clusters we investigated, Ni_19_ exhibits the highest stability and the strongest adsorption capability, making it a promising candidate for gas sensing and catalytic applications. Focusing here on a representative set of atmospheric and environmentally relevant gases, future studies may extend this analysis to H_2_S, which is of particular interest in sulfur poisoning and desulfurization chemistry.

Considering that gas adsorption is often the initial step in catalysis and sensing, our results provide first evidence on which Ni clusters can be more effective materials for these applications. A more detailed understanding of the involved processes would require investigation of activation mechanisms and reaction pathways through transition-state searches. Furthermore, exploring Ni cluster–gas interactions under real-life conditions, including condensed phases and finite temperatures *via* atomistic molecular dynamics simulations combined with machine-learned potentials parametrized on *ab initio* data, would provide deeper insight into their behavior.

## Author contributions

Mohsen Doust Mohammadi: project conceptualization, design, writing manuscript, investigation, analysis and resources, proofreading; Nikolaos Patsalidis: revision, review; Somnath Bhowmick: revision, review; Vagelis A. Harmandaris: revision, review; George Biskos: project conceptualization, supervision, revision, review.

## Conflicts of interest

There are no conflicts to declare.

## Supplementary Material

RA-016-D6RA01586G-s001

## Data Availability

The supporting data has been provided as part of the supplementary information (SI). Supplementary information is available. See DOI: https://doi.org/10.1039/d6ra01586g.
