# Supplementary material for: Structural, electronic, and gas adsorption properties of Nin (n = 1–20) atomic clusters
Source: RSC Adv. 2026 Apr 23;16(23):21372–80. doi: 10.1039/d6ra01586g (PMC13103942; doi:10.1039/d6ra01586g)
Supplement: RA-016-D6RA01586G-s001 [file RA-016-D6RA01586G-s001.pdf]

Supplementary Information (SI) for RSC Advances.
This journal is © The Royal Society of Chemistry 2026

Supporting Information for:

Structural, Electronic, and Gas Adsorption Properties of Ni_n (n = 1-20) Atomic Clusters

Mohsen Doust Mohammadi,[a] Nikolaos Patsalidis,[b] Somnath Bhowmick,[a]
Vagelis A. Harmandaris,[b, c, d] and George Biskos*[a, e]

[a]*Climate and Atmosphere Research Centre, The Cyprus Institute, Nicosia 2121, Cyprus*
[b]*Computation-based Science and Technology Research Centre, The Cyprus Institute, Nicosia 2121, Cyprus*
[c]*Institute of Applied and Computational Mathematics (IACM), Foundation for Research and Technology Hellas, (FORTH), IACM/FORTH, GR-71110 Heraklion, Crete, Greece*
[d]*Department of Mathematics and Applied Mathematics, University of Crete, GR-71409, Heraklion, Crete, Greece*
[e]*Faculty of Civil Engineering and Geosciences, Delft University of Technology, Delft 2628 CN, The Netherlands*

Conceptual DFT descriptors:

The electrophilicity index (ω) of the Ni_n system was evaluated within the conceptual density functional theory (DFT) framework to quantify its global electron-accepting capability. Electrophilicity reflects the stabilization energy of a system when it acquires additional electronic charge from the environment. It is computed from the electronic chemical potential (μ) and the chemical hardness (η), which are derived from frontier molecular orbital energies. In practice, μ and η are approximated using the energies of the highest occupied molecular orbital (E_{HOMO}) and the lowest unoccupied molecular orbital (E_{LUMO}). A lower chemical potential indicates a stronger tendency to accept electrons, while higher hardness corresponds to resistance to charge transfer. The electrophilicity index combines these quantities to provide a single descriptor of reactivity, allowing comparison of electrophilic character across related systems.

$$\mu = \frac{E_{\text{HOMO}} + E_{\text{LUMO}}}{2} \tag{1}$$

$$\eta = \frac{E_{\text{LUMO}} - E_{\text{HOMO}}}{2} \tag{2}$$

$$\omega = \frac{\mu^2}{2\eta} \tag{3}$$

Natural Population Analysis:

Natural Population Analysis (NPA) is a quantum chemical method designed to provide a physically meaningful description of electron distribution within molecules. It is based on the concept of Natural Atomic Orbitals (NAOs), which form an orthonormal set of atomic-like orbitals optimized to represent the electronic density of a molecule. The main advantage of NPA over traditional population analyses, such as Mulliken or Löwdin methods, is its reduced sensitivity to basis set choice and its ability to avoid unphysical charge delocalization.

In NPA, the one-particle density matrix **P** is expressed in the NAO basis. Each atomic orbital is associated with a specific atom, allowing electron populations to be projected directly onto individual atoms. The electron population q_A on atom A is calculated by summing over all occupied molecular orbitals ϕ_i, weighted by their projection onto the NAOs of atom A

$$q_A = \sum_i^{occ} \langle \phi_i | \hat{P}_A | \phi_i \rangle \tag{4}$$

Here, P_A represents the projection operator corresponding to the NAOs localized on atom A. This population q_A represents the total electronic density assigned to atom A. The atomic charge Q_A is then determined as the difference between the nuclear charge Z_A and the electron population:

$$Q_A = Z_A - q_A \tag{5}$$

This approach allows NPA to partition the total electron density of a molecule into contributions from each atom, providing insights into charge transfer, polarization, and electron delocalization. In addition, NPA can be extended to calculate bond orders and natural bond orbital (NBO) interactions, which quantify donor-acceptor interactions and orbital hybridization effects. NPA is widely used in computational chemistry for its accuracy in estimating atomic charges, its chemical interpretability, and its ability to support analyses of reactivity, electrophilicity, and molecular interactions. By examining the natural populations of electrons on atoms or functional groups, one can identify electron-rich and electron-deficient regions, which are critical for understanding chemical reactivity and noncovalent interactions.

Table S1

The values of Gupta potential including A_{ij} (eV), ξ_{ij} (eV), d_{ij}, p_{ij}, and q_{ij}

A_{ij} (eV)	ξ_{ij} (eV)	d_{ij}	p_{ij}	q_{ij}
0.038	1.07	2.491	16.999	1.189

Table S2

Cartesian co-ordinates of Ni_n clusters, with n = 2-20 calculated using the B3LYP-GD3BJ functional with the LANL2DZ basis set.

Ni_2

Ni 0.000000 0.000000 2.095000
Ni 0.000000 0.000000 0.000000

Ni_3

Ni -1.111041 0.641456 0.000000
Ni 1.111041 0.641461 0.000000
Ni 0.000000 -1.282917 0.000000

Ni_4

Ni 0.614775 -1.123043 -0.666434
Ni 1.123052 0.614777 0.666426
Ni -1.123042 -0.614781 0.666433
Ni -0.614785 1.123048 -0.666425

Ni_5

Ni 0.000080 0.000259 1.284785
Ni -0.284194 -1.142289 -0.696965
Ni 0.284082 1.142017 -0.697427
Ni -1.961952 0.165417 0.054817
Ni 1.961984 -0.165403 0.054791

Ni_6

Ni 0.268405 0.001121 1.230181
Ni -1.723370 -1.117776 0.025388
Ni -1.722632 1.118086 0.024962
Ni 0.434440 1.166276 -0.724259
Ni 0.433779 -1.167311 -0.722893
Ni 2.309377 -0.000395 0.166621

Ni_7

Ni 2.181224 0.805161 -0.377165
Ni 0.000002 1.506095 0.000006
Ni -2.181223 0.805166 0.377159
Ni 0.328839 -0.429609 -1.172175
Ni -0.328841 -0.429612 1.172173
Ni -1.799137 -1.128601 -0.671506

Ni 1.799135 -1.128600 0.671508

Ni_8

Ni -0.771104 0.001382 1.262432
Ni 1.250976 0.000875 -1.144027
Ni 1.231497 2.088851 -0.031326
Ni 1.238719 -2.085880 -0.031610
Ni -0.882607 -1.316434 -0.548332
Ni 1.538109 0.002100 1.021953
Ni -0.886610 1.312885 -0.552329
Ni -2.718981 -0.003778 0.023239

Ni_9

Ni 1.890584 -0.395681 -0.287887
Ni 0.058081 1.576475 -0.787727
Ni -0.849122 -1.459626 0.922483
Ni -0.388374 -0.879070 -1.170027
Ni -2.015073 0.574477 1.122000
Ni 0.400060 0.499997 1.158537
Ni -2.085632 0.703389 -1.084708
Ni 2.053640 1.861214 0.299590
Ni 0.935835 -2.481176 -0.172260

Ni_{10}

Ni -2.118553 0.317904 -1.045946
Ni 2.028237 -0.353555 0.576961
Ni -0.376127 -0.104855 1.197634
Ni -1.601385 1.731979 0.652236
Ni -1.718515 -1.630719 0.168593
Ni 0.057054 -1.021984 -1.095637
Ni 0.139380 1.416801 -0.832529
Ni 1.984050 0.221908 -1.560343
Ni 0.502091 -2.294031 0.776706
Ni 1.103768 1.716552 1.162324

Ni_{11}

Ni -1.748580 -1.300062 -1.283622
Ni -2.623085 -0.325676 0.544809
Ni -0.463031 -0.372301 1.205769
Ni 1.302054 1.191508 0.623727
Ni -0.745328 1.926115 1.432372
Ni -1.301247 1.192175 -0.623960
Ni 0.746778 1.925105 -1.432860
Ni 2.622792 -0.327304 -0.545091
Ni 1.747661 -1.300268 1.283863
Ni -0.000627 -2.236234 0.000453

Ni 0.462612 -0.373059 -1.205459

Ni_{12}

Ni 0.527418 0.609687 -1.125901
Ni 1.435231 -1.213950 0.509784
Ni 1.780623 1.607931 1.263651
Ni -0.143039 0.322548 1.425346
Ni -0.472344 -1.972574 1.831413
Ni -0.785540 -1.418147 -0.488368
Ni -2.178251 -0.507400 1.026894
Ni -2.325956 1.330708 -0.389313
Ni -0.266703 2.230294 0.232612
Ni -1.468216 0.091831 -2.066968
Ni 1.210378 -1.477885 -1.773347
Ni 2.686398 0.396958 -0.445804

Ni_{13}

Ni -1.733272 1.715992 -0.853370
Ni 0.004548 0.351454 -1.085792
Ni 2.253024 -1.266513 0.757406
Ni 1.744052 1.711692 -0.842180
Ni 0.002690 2.724070 0.362527
Ni 1.203090 0.696389 1.140789
Ni -0.005217 -1.564236 0.534124
Ni 0.002379 -1.921363 -1.702895
Ni 2.078785 -0.639329 -1.403022
Ni -2.072278 -0.634317 -1.410713
Ni -2.263731 -1.257872 0.749670
Ni -0.007512 -0.616657 2.619216
Ni -1.206557 0.700690 1.134242

Ni_{14}

Ni -2.006912 0.369319 -0.000315
Ni -1.957734 -1.672955 1.148958
Ni -0.000161 -0.181810 -1.371744
Ni -1.957937 -1.673333 -1.148902
Ni -1.364780 1.608858 -1.823555
Ni 1.365072 1.608307 -1.823506
Ni 0.000094 2.027615 0.000282
Ni 1.365111 1.608244 1.823792
Ni -1.365256 1.607943 1.823693
Ni 0.000146 -0.182098 1.371838
Ni 1.957830 -1.673474 1.148692
Ni 1.957636 -1.672997 -1.149254
Ni -0.000058 -2.142834 -0.000125
Ni 2.006951 0.369214 0.000147

Ni_{15}

Ni 2.271223 1.265474 -0.000180
Ni 2.445014 -0.395190 1.608332
Ni 0.278973 0.177037 -1.385059
Ni 0.144196 -1.837871 2.069161
Ni -2.046195 -0.426936 -1.643107
Ni -3.453346 0.273546 0.023752
Ni 0.146245 2.645687 1.066824
Ni 0.361962 2.536498 -1.171027
Ni 2.476097 -0.397841 -1.565058
Ni 1.374813 -1.628551 0.023785
Ni -1.957360 -0.311467 1.695044
Ni -1.058399 -1.563466 0.052624
Ni 0.094365 -2.004565 -1.959990
Ni 0.284660 0.300104 1.310897
Ni -1.362248 1.367539 -0.125999

Ni_{16}

Ni 1.187327 -0.507374 -1.978577
Ni 1.495239 2.119496 0.673418
Ni 2.689014 0.875341 -0.853254
Ni -1.624242 -0.500040 1.921694
Ni -1.766071 2.133388 -1.180953
Ni -2.814049 0.181591 0.055594
Ni 0.510295 1.772334 -1.420031
Ni -0.375711 -2.225854 -2.101827
Ni 0.293455 -1.869816 2.027375
Ni 0.152876 0.027418 0.216048
Ni 0.447986 0.718808 2.528762
Ni -1.042598 1.768608 0.960667
Ni 1.114135 -2.053707 -0.290498
Ni -1.356865 -1.797710 0.092370
Ni -1.184830 -0.052515 -1.715426
Ni 2.274038 -0.589968 1.064638

Ni_{17}

Ni -1.877833 -0.376589 2.274201
Ni 0.434943 2.891457 -0.000021
Ni -1.877657 -0.376698 -2.274284
Ni 1.393421 1.536031 -2.007864
Ni -1.514162 -0.644718 -0.000026
Ni -0.773750 -2.524487 1.235242
Ni -0.923467 1.525996 1.336632
Ni 1.393223 1.536064 2.007962
Ni -0.773716 -2.524470 -1.235260

Ni -2.761405 1.267598 -0.000139
Ni 0.936216 0.595783 0.000041
Ni 1.153343 -1.982339 0.000020
Ni 2.677045 -0.621968 1.165387
Ni -0.923344 1.525959 -1.336743
Ni 0.379912 -0.602837 1.922676
Ni 0.380089 -0.602853 -1.922646
Ni 2.677143 -0.621928 -1.165177

Ni_{18}

Ni -1.950066 2.265872 -0.232354
Ni -1.721111 1.097569 2.221153
Ni -1.869565 -1.884564 1.486697
Ni -1.989540 -1.834775 -1.134393
Ni -1.753268 0.601603 -2.176411
Ni -2.171460 0.017990 0.260164
Ni 0.040060 1.924492 0.986900
Ni -0.027774 -0.510693 2.053458
Ni -0.048029 -2.250992 0.099695
Ni -0.012999 -1.089237 -2.288643
Ni 0.045058 1.883845 -1.508292
Ni 0.003609 -0.031797 -0.295121
Ni 1.742366 1.009905 2.243838
Ni 1.774625 -1.942933 1.504141
Ni 1.922437 -1.915284 -1.111715
Ni 1.795758 0.528165 -2.162667
Ni 2.045808 2.192329 -0.222152
Ni 2.174091 -0.061493 0.275703

Ni_{19}

Ni 0.000190 -0.000118 0.000344
Ni -1.820360 1.735743 -0.006335
Ni -1.824625 -1.731853 0.007773
Ni 1.819571 -1.740200 0.011582
Ni 1.823948 1.735918 -0.013500
Ni -3.384202 0.004525 0.002632
Ni -0.008350 -3.253005 0.018353
Ni 3.383124 -0.004490 -0.001776
Ni 0.001447 3.252588 -0.018512
Ni -1.820401 0.014990 1.737365
Ni 1.823020 0.005218 1.732324
Ni 0.007337 1.677199 1.657915
Ni -0.005505 -1.658623 1.676905
Ni 0.006149 0.018827 3.251621
Ni -1.822813 -0.011086 -1.736191
Ni 1.821000 -0.009084 -1.734387

Ni -0.003283 1.657972 -1.676490
Ni 0.002052 -1.677009 -1.657689
Ni 0.001700 -0.017514 -3.251932

Ni_{20}
Ni 1.415878 2.297656 -0.177699
Ni 2.600836 -2.287271 -0.906923
Ni -1.586488 -2.664602 0.883050
Ni 2.633808 -1.337859 1.198477
Ni -0.830109 2.606765 -1.361247
Ni 1.423914 1.334279 -2.320816
Ni -1.403070 -1.566796 -1.213436
Ni 0.108903 -1.560228 2.467027
Ni -3.357178 -1.026446 -0.135405
Ni -0.985583 0.067468 -2.837032
Ni 0.990825 2.579935 2.087014
Ni 0.499936 -1.733385 0.265961
Ni 1.013781 0.422437 1.393763
Ni 2.376091 0.094256 -0.583947
Ni -1.553679 -0.382673 1.102738
Ni -0.092305 0.524958 -0.813529
Ni -0.992773 2.005109 0.922656
Ni -0.689153 0.901530 2.928748
Ni 0.854908 -1.247532 -2.097659
Ni -2.428543 0.972398 -0.801742

Table S3
Zero-point energies (ZPE, in eV) and ZPE-corrected electronic energies (EE, in eV) for the various structural isomers of neutral nickel clusters Ni_n (n = 1–20). For each n, individual isomers are labeled using the suffix "_01", "_02", and so on. All calculations were performed using the B3LYP-GD3BJ functional with the LANL2DZ basis set.

Systems	ZPE (eV)	EE (eV)
**Ni01**		-4614.2171
**Ni02**	0.021845	-9231.3679
**Ni03**	0.050317	-13848.964
**Ni04_01**	0.075216	-18465.886
**Ni04_03**	0.075244	-18465.886
**Ni04_02**	0.081707	-18465.718
**Ni05_01**	0.103252	-23083.182
**Ni05_02**	0.109661	-23082.914
**Ni06_01**	0.144897	-27700.716
**Ni06_02**	0.137179	-27700.569
**Ni06_03**	0.14686	-27700.511

**Ni07_01**	0.175551	-32318.335
**Ni07_03**	0.175087	-32318.302
**Ni07_02**	0.169605	-32318.224
**Ni07_04**	0.16126	-32318.145
**Ni08_05**	0.233995	-36936.084
**Ni08_09**	0.232222	-36936.082
**Ni08_06**	0.266721	-36936.015
**Ni08_08**	0.207514	-36936.001
**Ni08_07**	0.197914	-36935.916
**Ni08_04**	0.201132	-36935.914
**Ni08_12**	0.230177	-36935.866
**Ni08_03**	0.194205	-36935.863
**Ni08_01**	0.199496	-36935.846
**Ni08_13**	0.197150	-36935.842
**Ni08_10**	0.235958	-36935.649
**Ni08_11**	0.219568	-36935.625
**Ni08_02**	0.249049	-36935.561
**Ni09_06**	0.254967	-41553.952
**Ni09_04**	0.255622	-41553.951
**Ni09_03**	0.305911	-41553.892
**Ni09_02**	0.250576	-41553.817
**Ni09_01**	0.232522	-41553.801
**Ni09_13**	0.232658	-41553.801
**Ni09_09**	0.222922	-41553.781
**Ni09_05**	0.245749	-41553.762
**Ni09_12**	0.252758	-41553.728
**Ni09_14**	0.246758	-41553.694
**Ni09_10**	0.243758	-41553.691
**Ni09_07**	0.234513	-41553.635
**Ni09_11**	0.237867	-41553.609
**Ni09_08**	0.263258	-41553.541
**Ni10_02**	0.274603	-46171.804
**Ni10_05**	0.288894	-46171.803
**Ni10_08**	0.314147	-46171.714
**Ni10_15**	0.282594	-46171.703
**Ni10_17**	0.338447	-46171.703
**Ni10_20**	0.297048	-46171.700
**Ni10_11**	0.299339	-46171.688
**Ni10_09**	0.268958	-46171.571
**Ni10_01**	0.267349	-46171.565
**Ni10_06**	0.267649	-46171.565
**Ni10_12**	0.263803	-46171.546
**Ni10_14**	0.263912	-46171.546

**Ni10_16**	0.274712	-46171.531
**Ni10_23**	0.307629	-46171.505
**Ni10_18**	0.298275	-46171.500
**Ni10_03**	0.274439	-46171.466
**Ni10_19**	0.284748	-46171.441
**Ni10_13**	0.276048	-46171.435
**Ni10_10**	0.268112	-46171.411
**Ni10_22**	0.265330	-46171.397
**Ni10_21**	0.281857	-46171.200
**Ni10_07**	0.327784	-46171.077
**Ni10_04**	0.286548	-46170.784
**Ni11_12**	0.306348	-50789.804
**Ni11_04**	0.332665	-50789.747
**Ni11_02**	0.318184	-50789.677
**Ni11_01**	0.308911	-50789.619
**Ni11_18**	0.300811	-50789.602
**Ni11_07**	0.341229	-50789.589
**Ni11_09**	0.361465	-50789.512
**Ni11_14**	0.327593	-50789.501
**Ni11_06**	0.298957	-50789.408
**Ni11_05**	0.339892	-50789.396
**Ni11_03**	0.301111	-50789.386
**Ni11_13**	0.306157	-50789.341
**Ni11_15**	0.303757	-50789.159
**Ni11_08**	0.303320	-50789.135
**Ni11_17**	0.366674	-50789.12
**Ni11_10**	0.309348	-50788.974
**Ni11_16**	0.302066	-50788.941
**Ni11_11**	0.299584	-50788.743
**Ni12_12**	0.338256	-55407.721
**Ni12_01**	0.372510	-55407.578
**Ni12_03**	0.341829	-55407.547
**Ni12_07**	0.329393	-55407.529
**Ni12_17**	0.328847	-55407.507
**Ni12_14**	0.355792	-55407.466
**Ni12_09**	0.323011	-55407.464
**Ni12_10**	0.337765	-55407.450
**Ni12_02**	0.351292	-55407.440
**Ni12_22**	0.344201	-55407.439
**Ni12_16**	0.364328	-55407.419
**Ni12_25**	0.336729	-55407.381
**Ni12_05**	0.337411	-55407.337
**Ni12_13**	0.380391	-55407.269

**Ni12_24**	0.359583	-55407.263
**Ni12_06**	0.338256	-55407.261
**Ni12_23**	0.344938	-55407.256
**Ni12_21**	0.373492	-55407.162
**Ni12_11**	0.367437	-55407.064
**Ni12_18**	0.366237	-55407.005
**Ni12_15**	0.365855	-55406.881
**Ni12_04**	0.333620	-55406.753
**Ni12_19**	0.370655	-55406.239
**Ni12_20**	0.329311	-55406.236
**Ni12_08**	0.408209	-55406.170
**Ni13_02**	0.367110	-60025.684
**Ni13_22**	0.366237	-60025.631
**Ni13_05**	0.390291	-60025.611
**Ni13_16**	0.372919	-60025.604
**Ni13_15**	0.376955	-60025.59
**Ni13_08**	0.369619	-60025.557
**Ni13_20**	0.370628	-60025.516
**Ni13_18**	0.365337	-60025.513
**Ni13_14**	0.372619	-60025.462
**Ni13_21**	0.380255	-60025.399
**Ni13_07**	0.400955	-60025.368
**Ni13_17**	0.530497	-60025.364
**Ni13_13**	0.393182	-60025.343
**Ni13_11**	0.370846	-60025.342
**Ni13_01**	0.373901	-60025.312
**Ni13_09**	0.392091	-60025.273
**Ni13_03**	0.376001	-60025.244
**Ni13_12**	0.448517	-60025.235
**Ni13_04**	0.396046	-60025.176
**Ni13_19**	0.379301	-60025.048
**Ni13_10**	0.379791	-60024.823
**Ni13_06**	0.372701	-60024.714
**Ni14_20**	0.402155	-64643.706
**Ni14_21**	0.402755	-64643.656
**Ni14_07**	0.411782	-64643.481
**Ni14_24**	0.403873	-64643.477
**Ni14_25**	0.426999	-64643.475
**Ni14_22**	0.412709	-64643.407
**Ni14_18**	0.429072	-64643.361
**Ni14_06**	0.402591	-64643.360
**Ni14_17**	0.402182	-64643.326
**Ni14_14**	0.420972	-64643.298

**Ni14_03**	0.409245	-64643.256
**Ni14_05**	0.494634	-64643.198
**Ni14_19**	0.435481	-64643.150
**Ni14_11**	0.406164	-64643.120
**Ni14_13**	0.403245	-64643.088
**Ni14_10**	0.409627	-64643.077
**Ni14_16**	0.454599	-64643.000
**Ni14_02**	0.395800	-64642.965
**Ni14_04**	0.428499	-64642.884
**Ni14_12**	0.395746	-64642.872
**Ni14_01**	0.392282	-64642.802
**Ni14_08**	0.392773	-64642.727
**Ni14_09**	0.407173	-64642.654
**Ni14_15**	0.427572	-64642.511
**Ni14_23**	0.430845	-64641.974
**Ni15_06**	0.486807	-69261.431
**Ni15_02**	0.436299	-69261.369
**Ni15_10**	0.436899	-69261.360
**Ni15_12**	0.421518	-69261.358
**Ni15_14**	0.455117	-69261.307
**Ni15_11**	0.470253	-69261.276
**Ni15_19**	0.489671	-69261.275
**Ni15_01**	0.451408	-69261.166
**Ni15_07**	0.451599	-69261.136
**Ni15_15**	0.427245	-69261.070
**Ni15_03**	0.522725	-69261.063
**Ni15_09**	0.433599	-69260.946
**Ni15_16**	0.427709	-69260.690
**Ni15_13**	0.478353	-69260.665
**Ni15_04**	0.425172	-69260.578
**Ni15_08**	0.446663	-69260.535
**Ni15_05**	0.444399	-69260.527
**Ni15_17**	0.450563	-69260.016
**Ni15_18**	0.436463	-69259.537
**Ni16_03**	0.575496	-73879.350
**Ni16_04**	0.485607	-73879.346
**Ni16_09**	0.537397	-73879.345
**Ni16_05**	0.467335	-73879.301
**Ni16_10**	0.554088	-73879.291
**Ni16_08**	0.466844	-73879.279
**Ni16_12**	0.559160	-73879.251
**Ni16_11**	0.511434	-73879.226
**Ni16_13**	0.489344	-73879.190

**Ni16_06**	0.550624	-73879.152
**Ni16_07**	0.525561	-73879.105
**Ni16_01**	0.699584	-73878.872
**Ni17_10**	0.516779	-78497.772
**Ni17_16**	0.516288	-78497.771
**Ni17_04**	0.515879	-78497.631
**Ni17_01**	0.514434	-78497.553
**Ni17_11**	0.556242	-78497.300
**Ni17_06**	0.492043	-78497.266
**Ni17_07**	0.492207	-78497.265
**Ni17_05**	0.515034	-78497.262
**Ni17_09**	0.544870	-78497.257
**Ni17_14**	0.544815	-78497.253
**Ni17_19**	0.515607	-78497.227
**Ni17_20**	0.495398	-78497.141
**Ni17_02**	0.489044	-78497.141
**Ni17_08**	0.593578	-78497.085
**Ni17_18**	0.488853	-78497.020
**Ni17_17**	0.505625	-78496.980
**Ni17_12**	0.578878	-78496.960
**Ni17_15**	0.629849	-78496.835
**Ni17_03**	0.629577	-78496.809
**Ni17_13**	0.805755	-78496.070
**Ni18_07**	0.616377	-83115.650
**Ni18_05**	0.524961	-83115.513
**Ni18_02**	0.571978	-83115.471
**Ni18_01**	0.651940	-83115.278
**Ni18_04**	0.689521	-83115.219
**Ni18_09**	0.595050	-83115.102
**Ni18_03**	0.567560	-83115.033
**Ni18_10**	0.707384	-83114.669
**Ni18_06**	0.597532	-83114.328
**Ni18_08**	0.591396	-83114.175
**Ni19_08**	0.578932	-87734.637
**Ni19_05**	0.574733	-87733.861
**Ni19_04**	0.634649	-87733.855
**Ni19_01**	0.638195	-87733.628
**Ni19_07**	0.617413	-87733.578
**Ni19_09**	0.659194	-87733.100
**Ni19_02**	0.572305	-87732.914
**Ni19_10**	0.675667	-87732.816
**Ni19_03**	0.646322	-87732.717
**Ni19_06**	0.580841	-87731.777

**Ni20_08**	0.598814	-92351.575
**Ni20_01**	0.598568	-92351.575
**Ni20_07**	0.685348	-92351.346
**Ni20_10**	0.713820	-92351.332
**Ni20_06**	0.691675	-92351.217
**Ni20_04**	0.653876	-92351.177
**Ni20_09**	0.701957	-92351.064
**Ni20_02**	0.756583	-92350.847
**Ni20_05**	0.806791	-92350.670
**Ni20_03**	0.638604	-92348.512

Table S4

The average bond length values in Å for global minimum structures of Ni_n (n = 2-20). All values are calculated at the B3LYP-GD3BJ/LANl2DZ level of theory.

**Systems**	**Average Bond Length**
Ni_2	2.095
Ni_3	2.222
Ni_4	2.352
Ni_5	2.360
Ni_6	2.357
Ni_7	2.335
Ni_8	2.364
Ni_9	2.407
Ni_{10}	2.441
Ni_{11}	2.432
Ni_{12}	2.411
Ni_{13}	2.454
Ni_{14}	2.414
Ni_{15}	2.452
Ni_{16}	2.467
Ni_{17}	2.467
Ni_{18}	2.452
Ni_{19}	2.413
Ni_{20}	2.466

Table S5

Values of Cohesive energy per atom (eV), first and second dissociation energy ($D_{e,1}$ and $D_{e,2}$) in eV of the global minimum structure of Ni_n clusters, where n = 2-20. All values are obtained from electronic energy at the B3LYP-GD3BJ/LANl2DZ level of theory. The calculated cohesive energies are compared with those reported by Chibani et al.

**Systems**	E_{Coh}	E_{Coh}**(Chibani)**	$D_{e,1}$	$D_{e,2}$
Ni_2	1.478	1.538	2.956	-
Ni_3	2.121	1.975	3.407	3.407
Ni_4	2.273	2.311	2.730	3.182
Ni_5	2.440	2.578	3.106	2.881
Ni_6	2.593	2.806	3.359	3.510
Ni_7	2.713	2.912	3.433	3.836
Ni_8	2.823	2.991	3.591	4.068
Ni_9	2.917	3.101	3.671	4.306
Ni_{10}	2.991	3.167	3.670	4.386
Ni_{11}	3.066	3.212	3.799	4.514
Ni_{12}	3.121	3.259	3.733	4.576
Ni_{13}	3.171	3.285	3.775	4.552
Ni_{14}	3.219	3.378	3.839	4.658
Ni_{15}	3.244	3.433	3.593	4.477
Ni_{16}	3.276	-	3.751	4.388
Ni_{17}	3.329	-	4.186	4.981
Ni_{18}	3.353	-	3.761	4.991
Ni_{19}	3.426	-	4.732	5.538
Ni_{20}	3.392	-	2.741	4.518

Table S6

The values of HOMO energy (ε_{HOMO}) in eV, LUMO energy (ε_{LUMO}) in eV, HOMO-LUMO energy gap (HLG) in eV, chemical hardness (η) in eV, chemical potential (μ) in eV, and electrophilicity index (ω) in eV for the most stable isomers of neutral Nikel atomic clusters, Ni_n (n = 1-20). All values are calculated at the B3LYP-GD3BJ/LANl2DZ level of theory

**Systems**	**ε_{HOMO} (eV)**	**ε_{LUMO} (eV)**	**HLG (eV)**	**μ (eV)**	**η (eV)**	**ω (eV)**
**Ni_1**	-4.8825	-3.2411	1.6414	-4.0618	0.8207	10.0513
**Ni_2**	-5.4001	-3.3522	2.0479	-4.3761	1.0240	9.3507
**Ni_3**	-4.4504	-2.2439	2.2066	-3.3471	1.1033	5.0771
**Ni_4**	-3.9141	-2.2082	1.7059	-3.0611	0.8529	5.4932
**Ni_5**	-4.4107	-2.8346	1.5761	-3.6227	0.7880	8.3274
**Ni_6**	-4.7680	-3.0210	1.7470	-3.8945	0.8735	8.6818
**Ni_7**	-4.4583	-2.8768	1.5815	-3.6676	0.7908	8.5049
**Ni_8**	-4.8643	-3.2776	1.5867	-4.0710	0.7933	10.4456
**Ni_9**	-4.7998	-3.3173	1.4825	-4.0586	0.7412	11.1119
**Ni_{10}**	-4.7985	-3.2251	1.5734	-4.0118	0.7867	10.2291
**Ni_{11}**	-4.7917	-3.3500	1.4417	-4.0708	0.7208	11.4952
**Ni_{12}**	-4.8502	-3.3933	1.4569	-4.1217	0.7284	11.6615
**Ni_{13}**	-4.7538	-3.3601	1.3938	-4.0569	0.6969	11.8083
**Ni_{14}**	-4.6817	-3.3356	1.3461	-4.0086	0.6731	11.9365
**Ni_{15}**	-4.7100	-3.2888	1.4213	-3.9994	0.7106	11.2547
**Ni_{16}**	-4.5707	-3.3108	1.2599	-3.9408	0.6299	12.3273
**Ni_{17}**	-4.5838	-3.2665	1.3173	-3.9251	0.6587	11.6946
**Ni_{18}**	-4.6488	-3.4001	1.2487	-4.0244	0.6244	12.9691
**Ni_{19}**	-4.3391	-3.2850	1.0542	-3.8120	0.5271	13.7842
**Ni_{20}**	-4.4959	-3.3269	1.1690	-3.9114	0.5845	13.0873

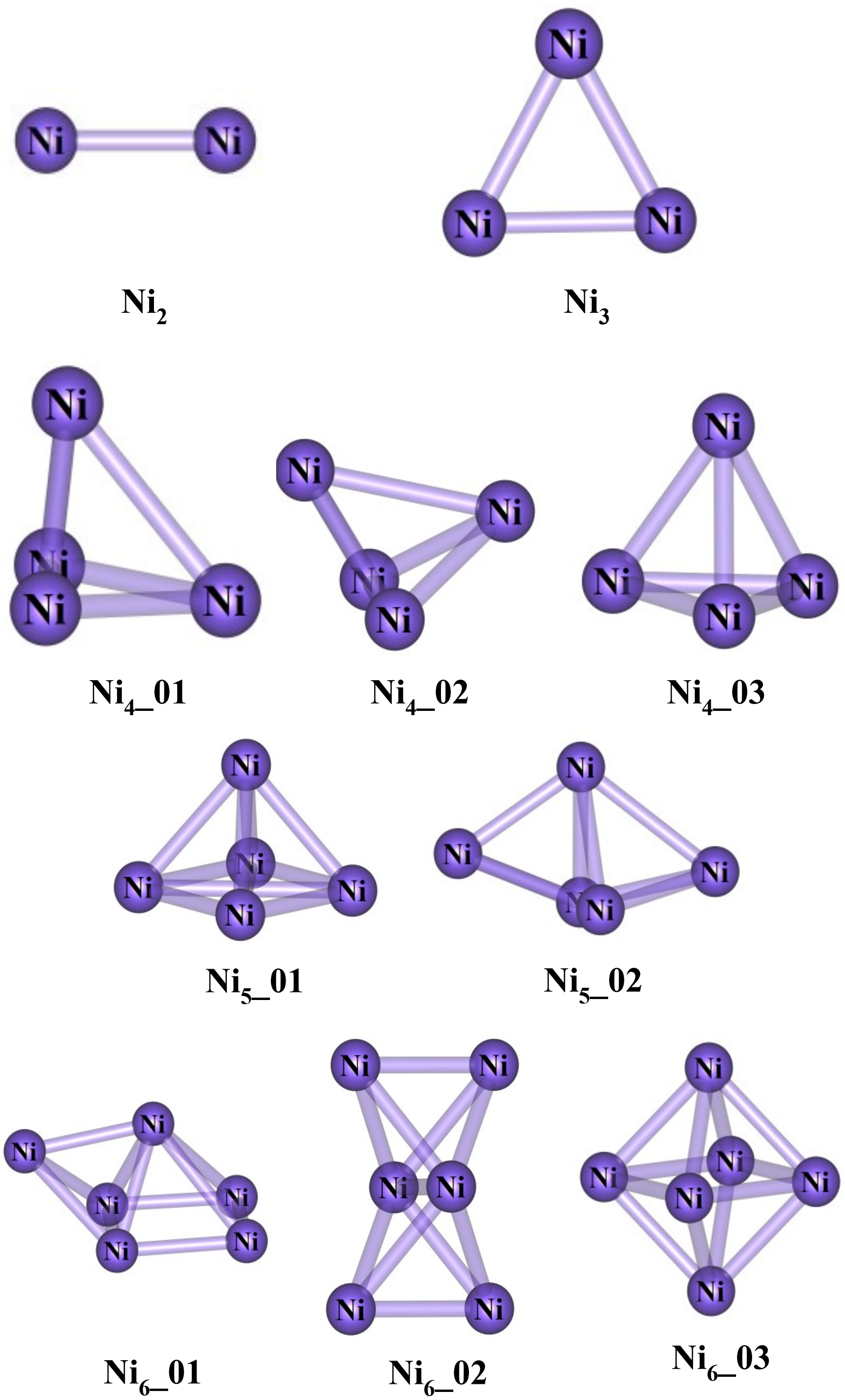

Figure S1. Illustration of nickel atomic clusters, Ni_n (n = 2-6), as determined through the geometric optimization process using the DFT method at B3LYP-GD3BJ/LANl2DZ. The X variable in Ni_n_X name shows different structural isomers for a specific cluster size.

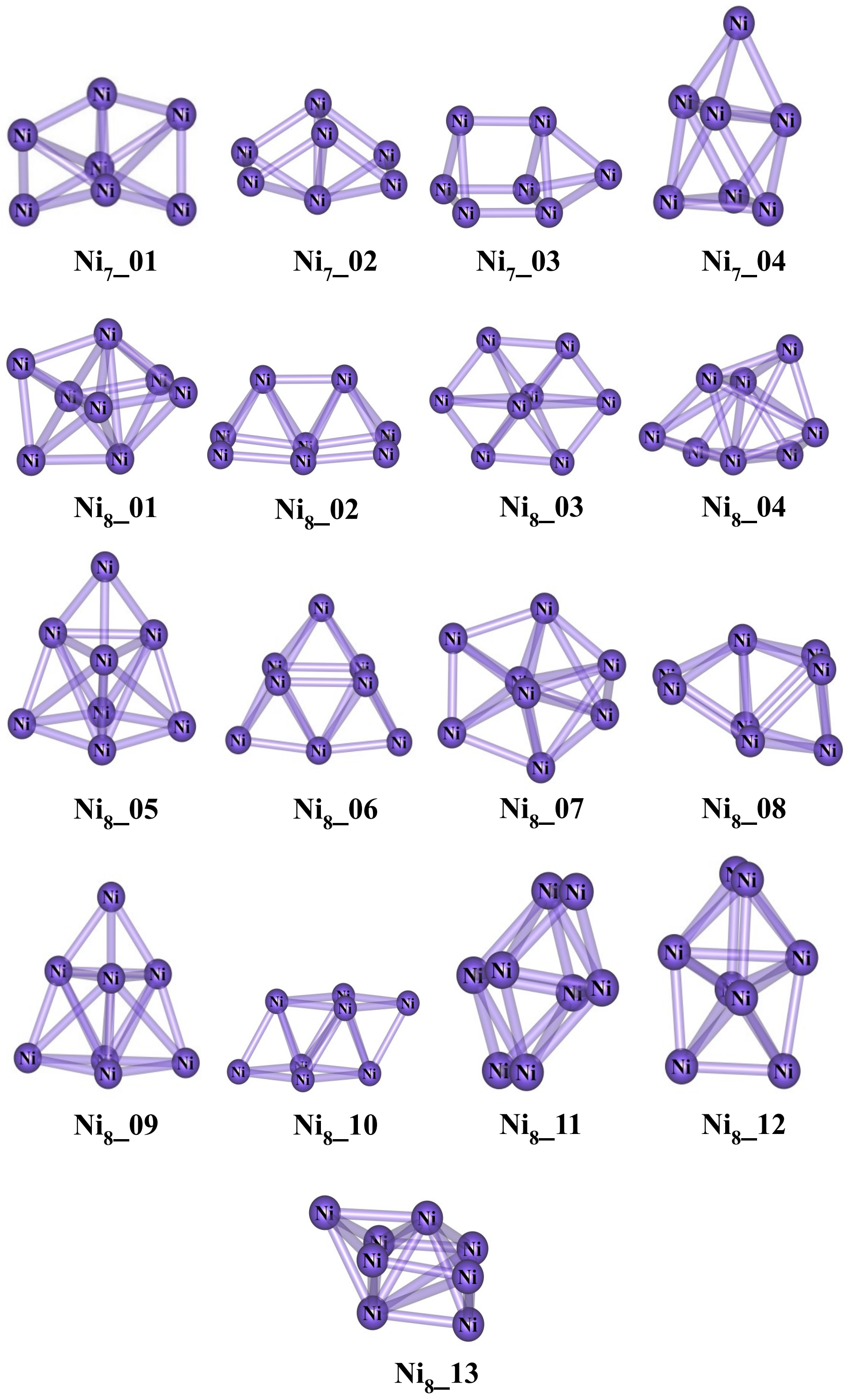

Figure S2. Illustration of nickel atomic clusters, Ni_n (n = 7 and 8), as determined through the geometric optimization process using the DFT method at B3LYP-GD3BJ/LANl2DZ. The X variable in Ni_n-X name shows different structural isomers for a specific cluster size.

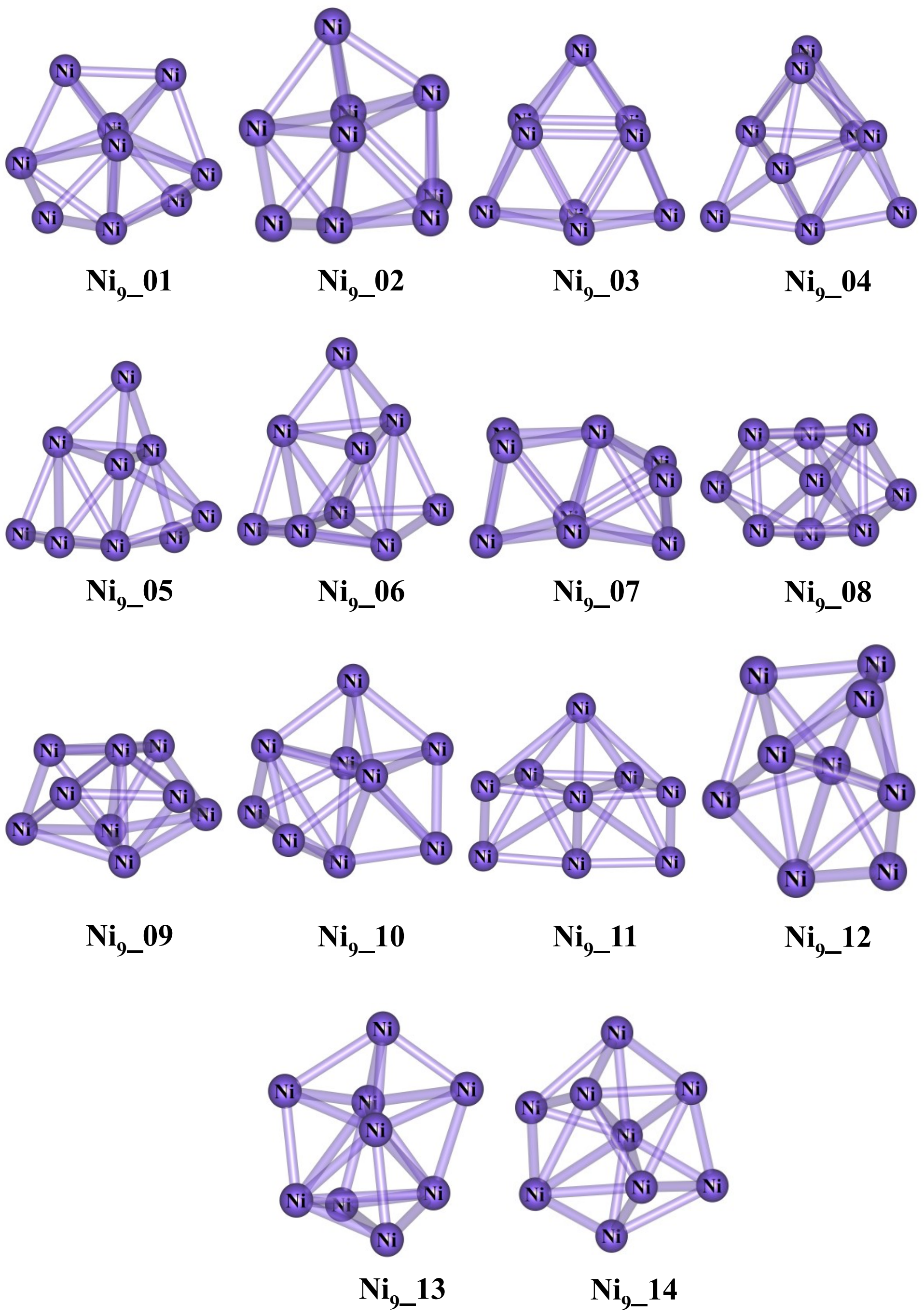

Figure S3. Illustration of nickel atomic clusters, Ni_9, as determined through the geometric optimization process using the DFT method at B3LYP-GD3BJ/LANl2DZ. The X variable in Ni_n_X name shows different structural isomers for a specific cluster size.

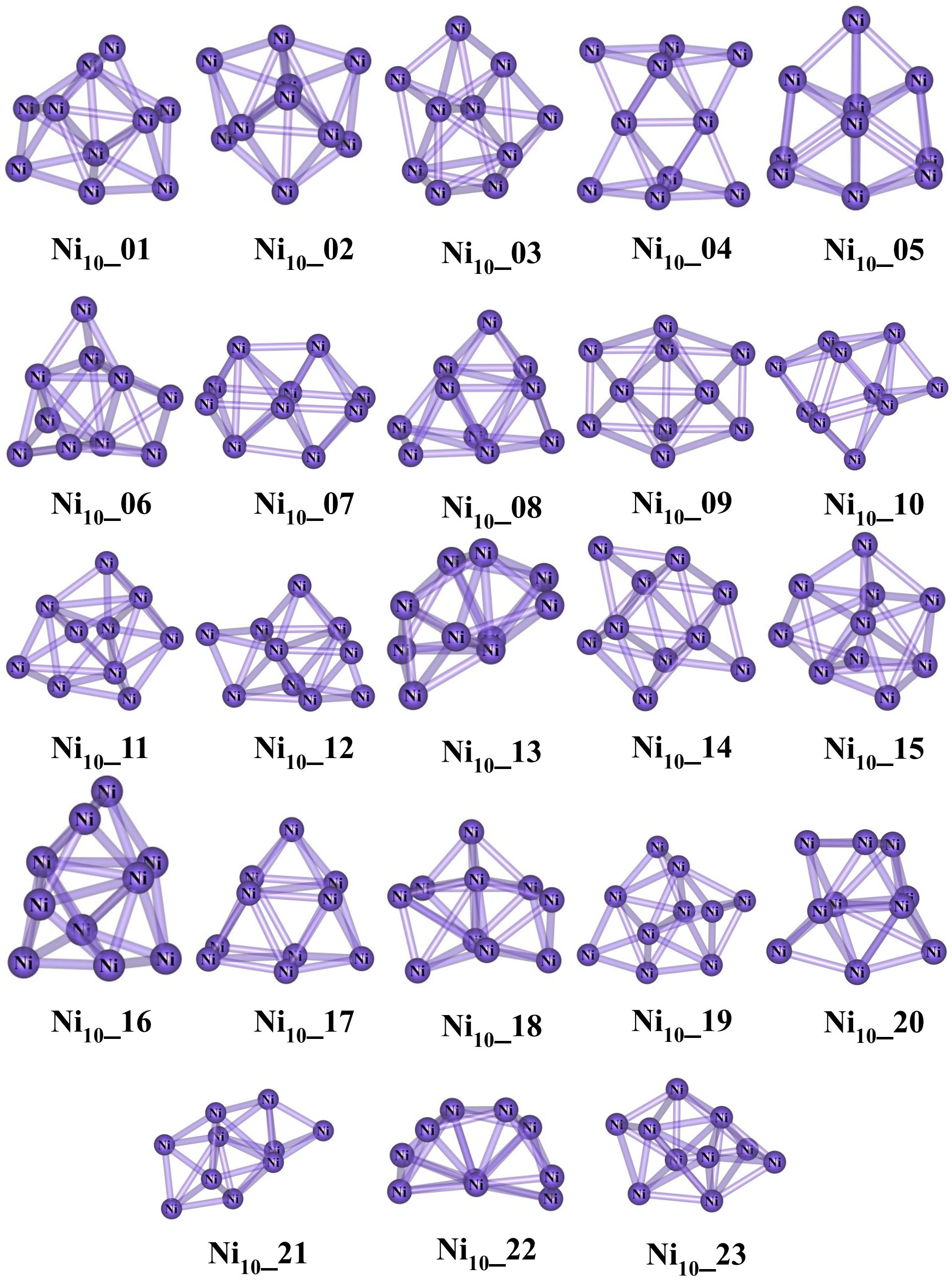

Figure S4. Illustration of nickel atomic clusters, Ni_{10}, as determined through the geometric optimization process using the DFT method at B3LYP-GD3BJ/LANl2DZ. The X variable in Ni_n_X name shows different structural isomers for a specific cluster size.

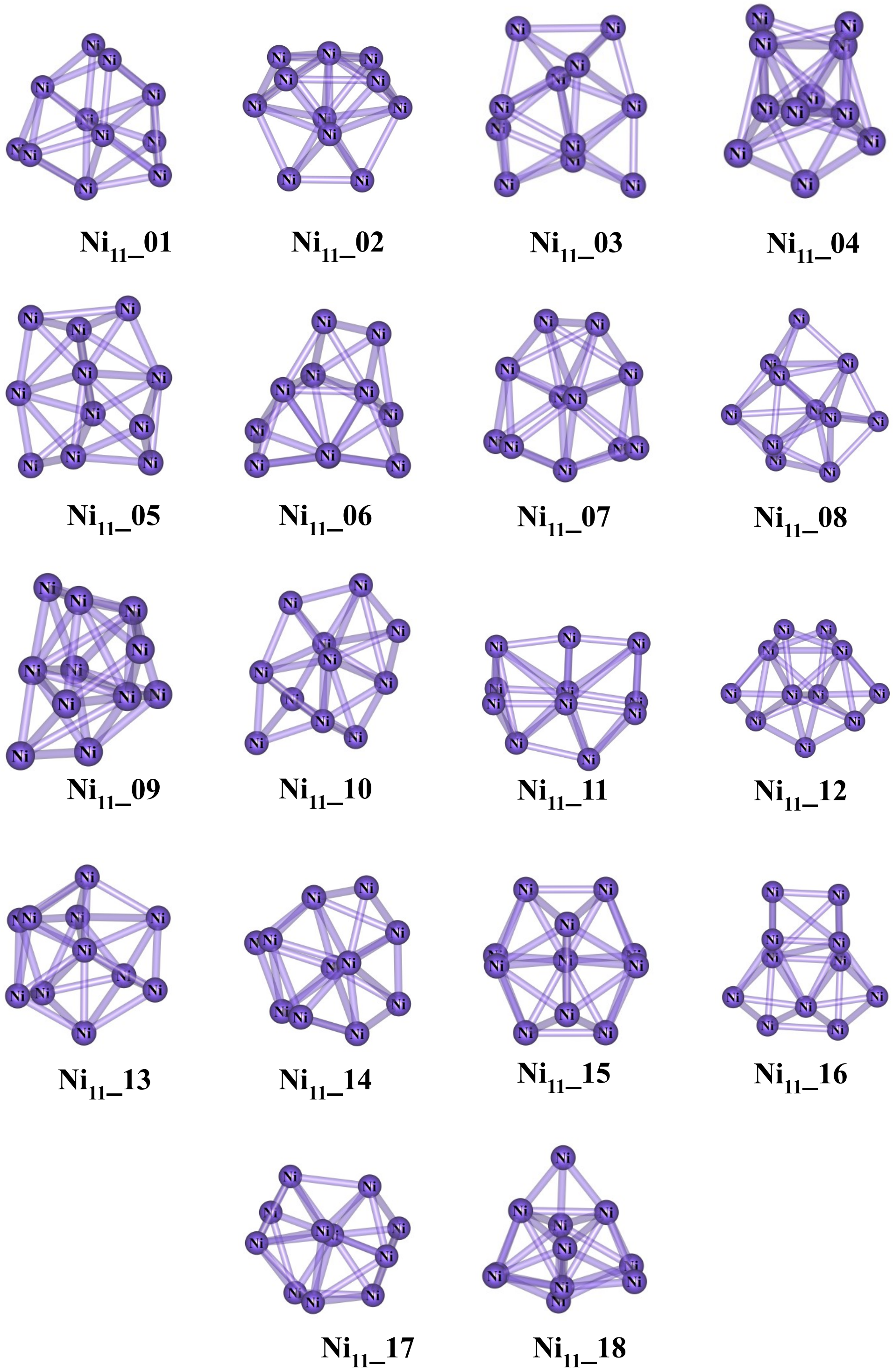

Figure S5. Illustration of nickel atomic clusters, Ni_{11}, as determined through the geometric optimization process using the DFT method at B3LYP-GD3BJ/LANl2DZ. The X variable in Ni_n_X name shows different structural isomers for a specific cluster size.

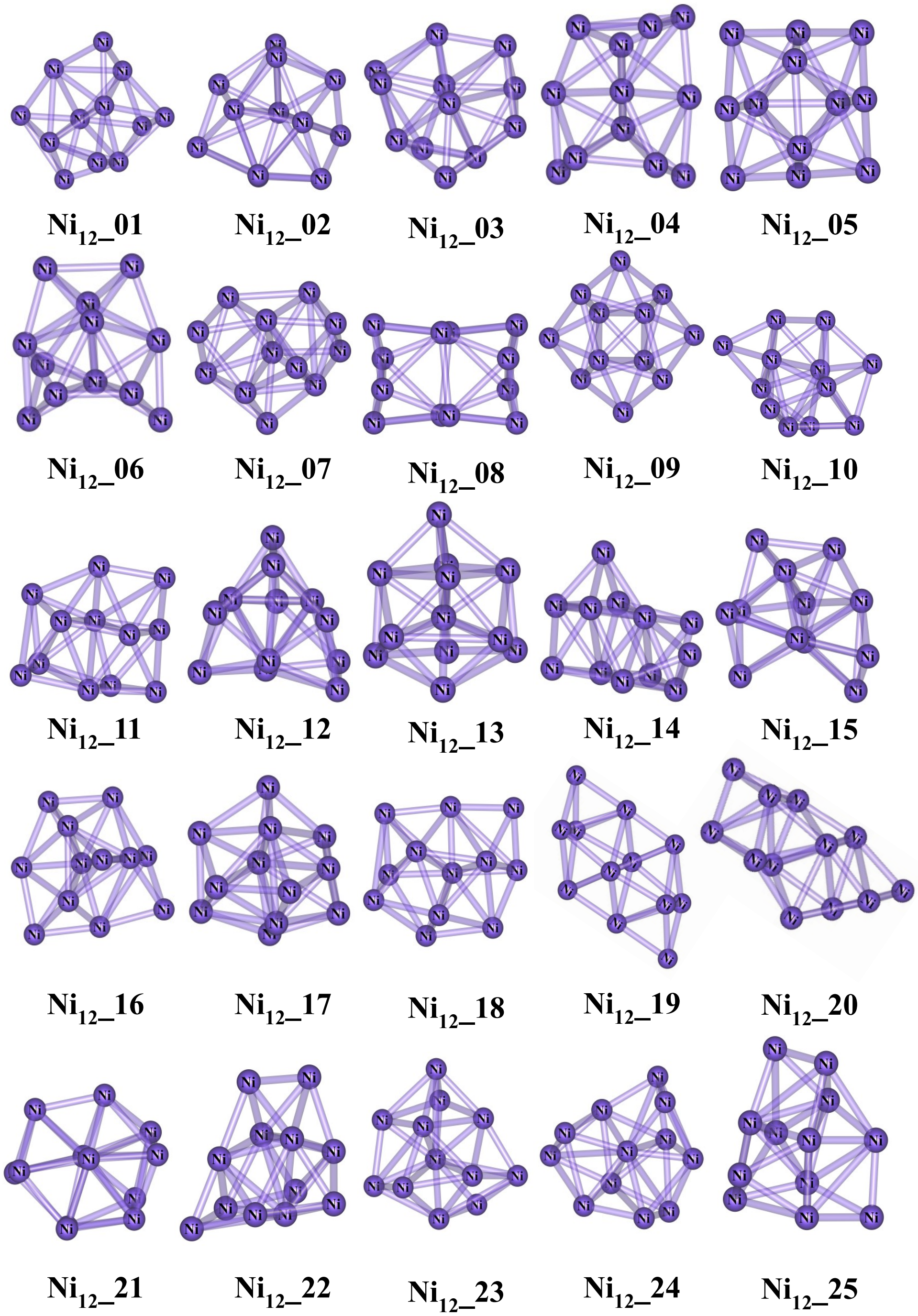

Figure S6. Illustration of nickel atomic clusters, Ni_{12}, as determined through the geometric optimization process using the DFT method at B3LYP-GD3BJ/LANl2DZ. The X variable in Ni_n_X name shows different structural isomers for a specific cluster size.

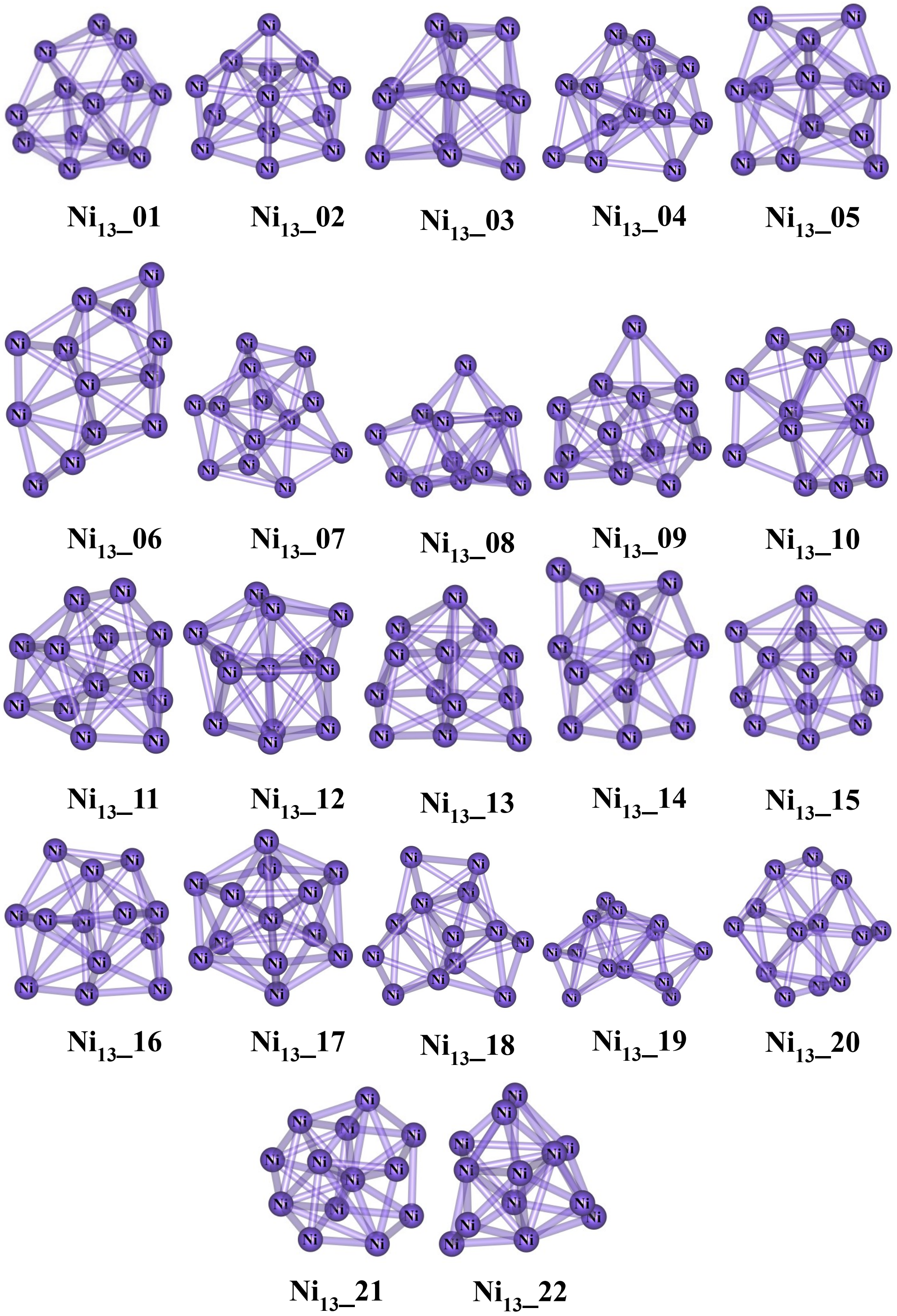

Figure S7. Illustration of nickel atomic clusters, Ni_{13}, as determined through the geometric optimization process using the DFT method at B3LYP-GD3BJ/LANl2DZ. The X variable in Ni_n_X name shows different structural isomers for a specific cluster size.

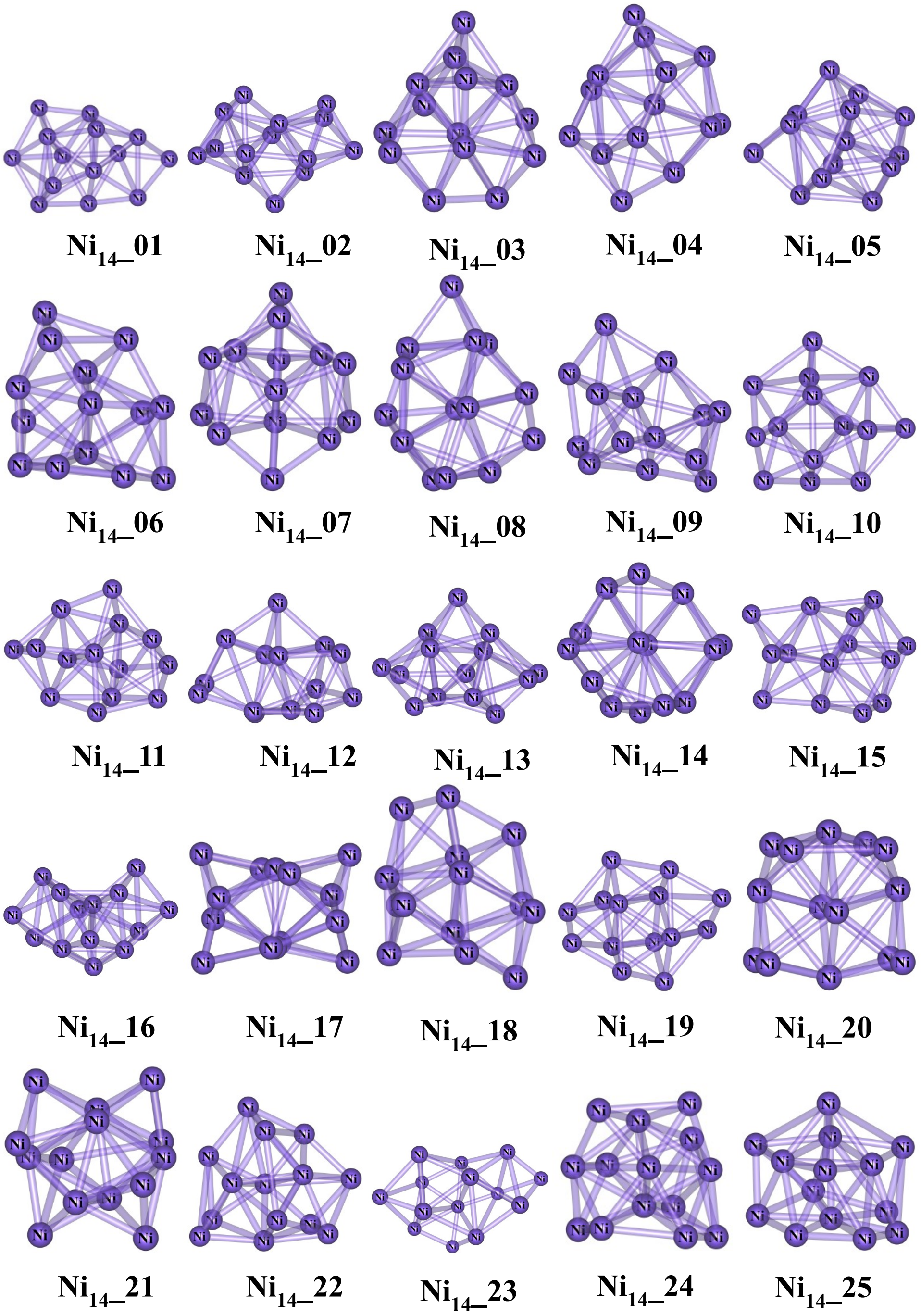

Figure S8. Illustration of nickel atomic clusters, Ni_{14}, as determined through the geometric optimization process using the DFT method at B3LYP-GD3BJ/LANl2DZ. The X variable in Ni_n_X name shows different structural isomers for a specific cluster size.

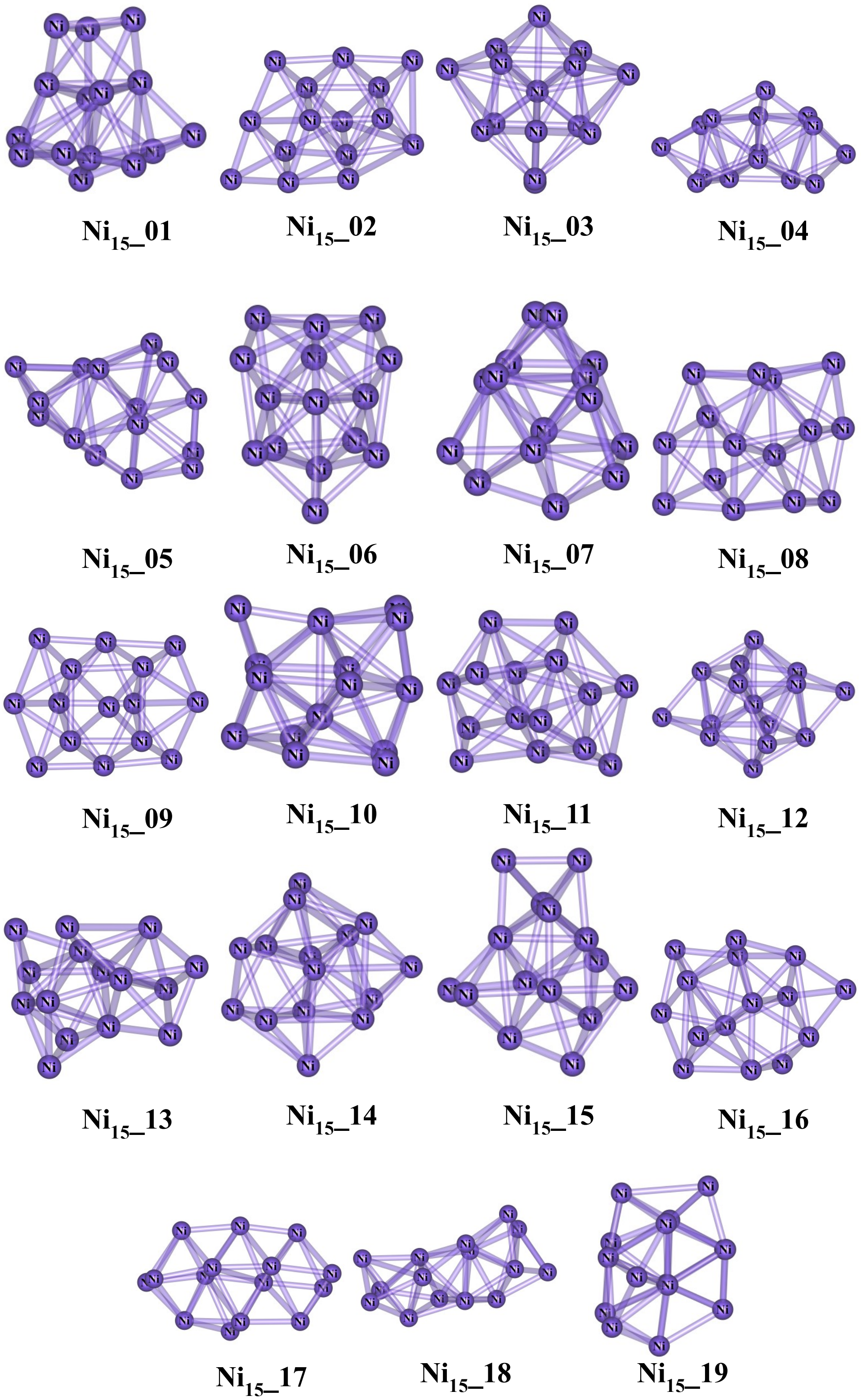

Figure S9. Illustration of nickel atomic clusters, Ni_{15}, as determined through the geometric optimization process using the DFT method at B3LYP-GD3BJ/LANl2DZ. The X variable in Ni_n_X name shows different structural isomers for a specific cluster size.

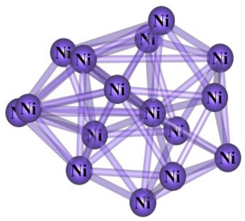

Ni_{16}_01

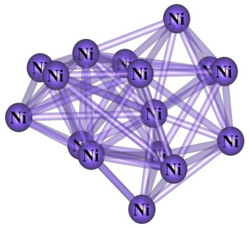

Ni_{16}_02

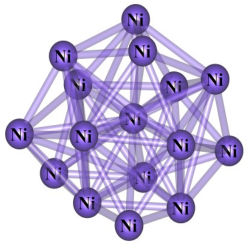

Ni_{16}_03

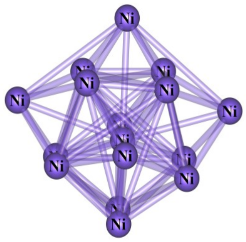

Ni_{16}_04

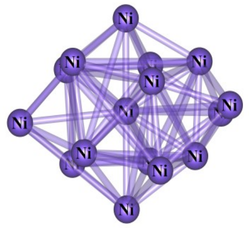

Ni_{16}_05

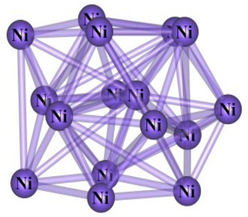

Ni_{16}_06

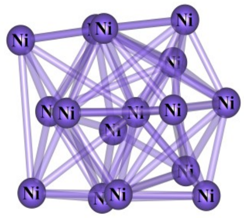

Ni_{16}_07

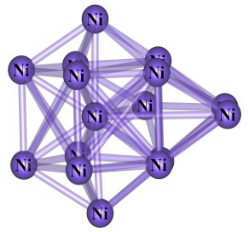

Ni_{16}_08

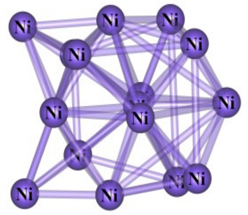

Ni_{16}_09

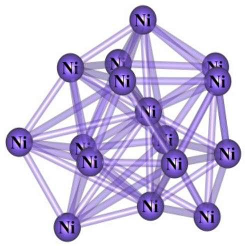

Ni_{16})10

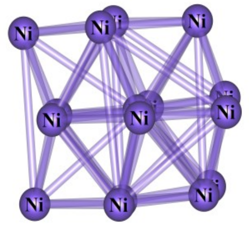

Ni_{16}_11

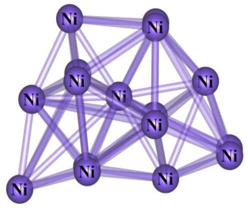

Ni_{16}_12

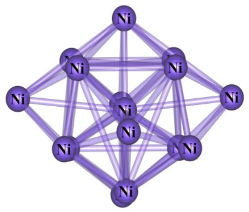

Ni_{16}_13

Figure S10. Illustration of nickel atomic clusters, Ni_{16}, as determined through the geometric optimization process using the DFT method at B3LYP-GD3BJ/LANl2DZ. The X variable in Ni_n_X name shows different structural isomers for a specific cluster size.

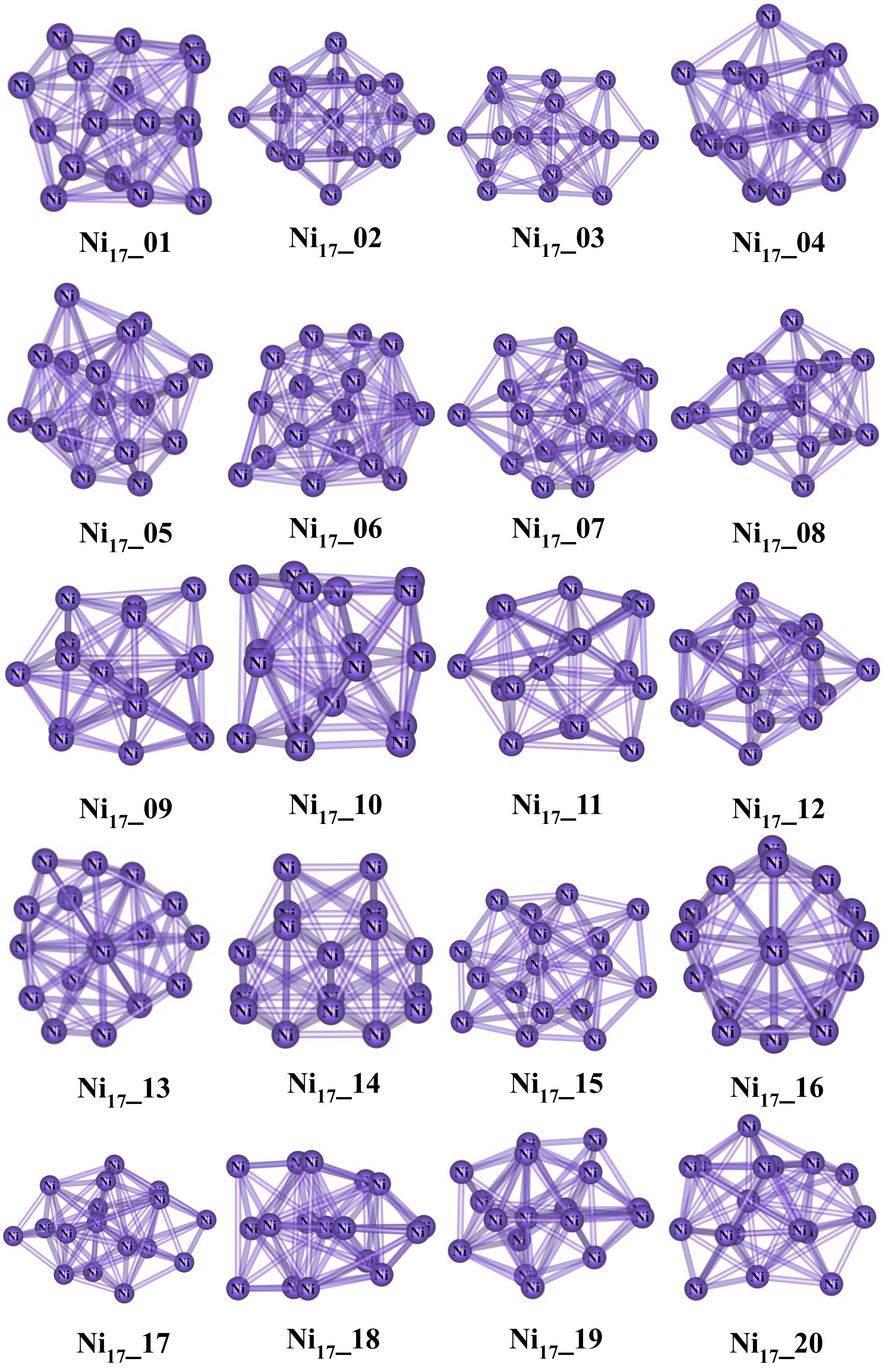

Figure S11. Illustration of nickel atomic clusters, Ni_{17}, as determined through the geometric optimization process using the DFT method at B3LYP-GD3BJ/LANl2DZ. The X variable in Ni_n_X name shows different structural isomers for a specific cluster size.

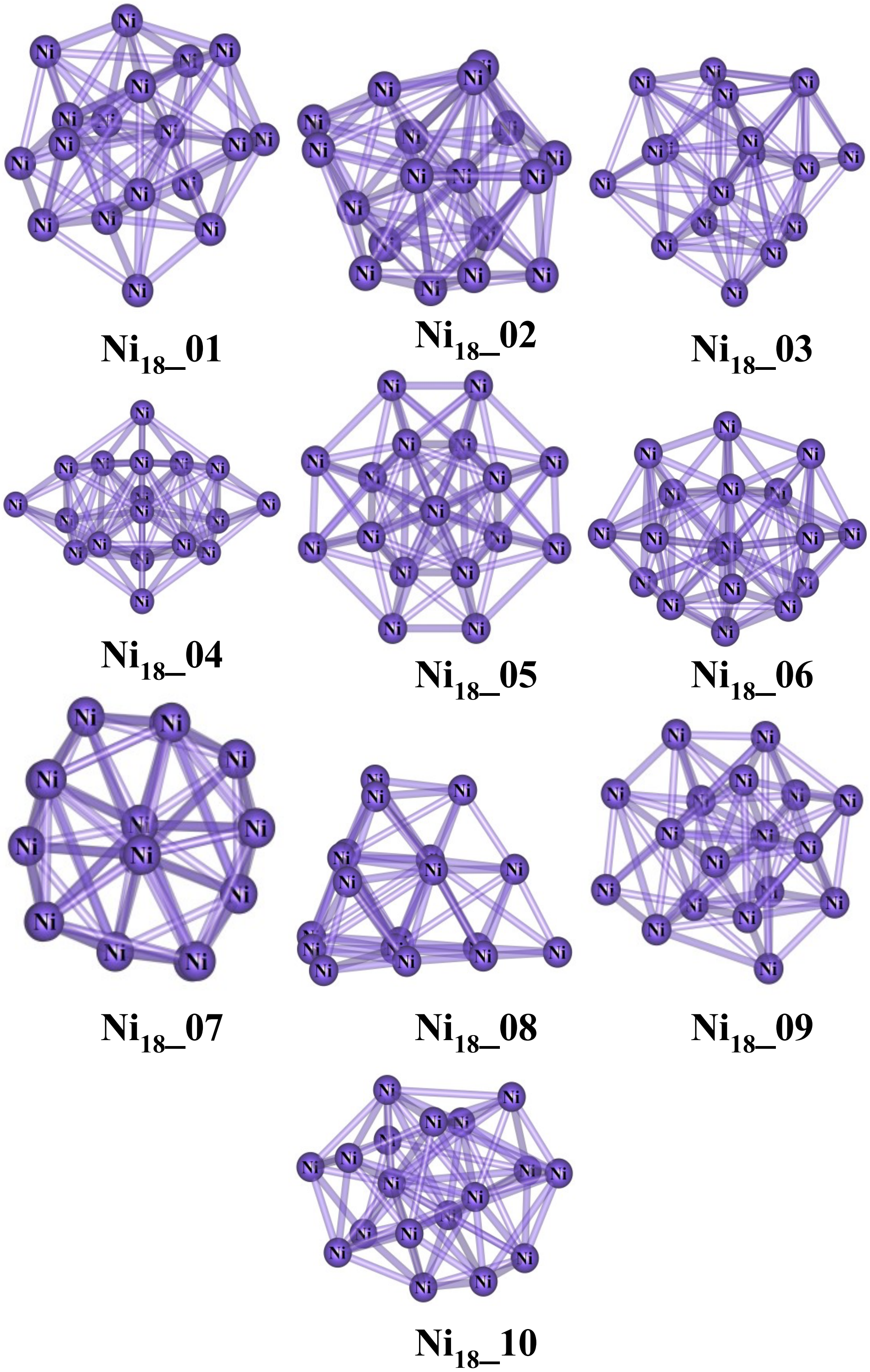

Figure S12. Illustration of nickel atomic clusters, Ni_{18}, as determined through the geometric optimization process using the DFT method at B3LYP-GD3BJ/LANl2DZ. The X variable in Ni_n_X name shows different structural isomers for a specific cluster size.

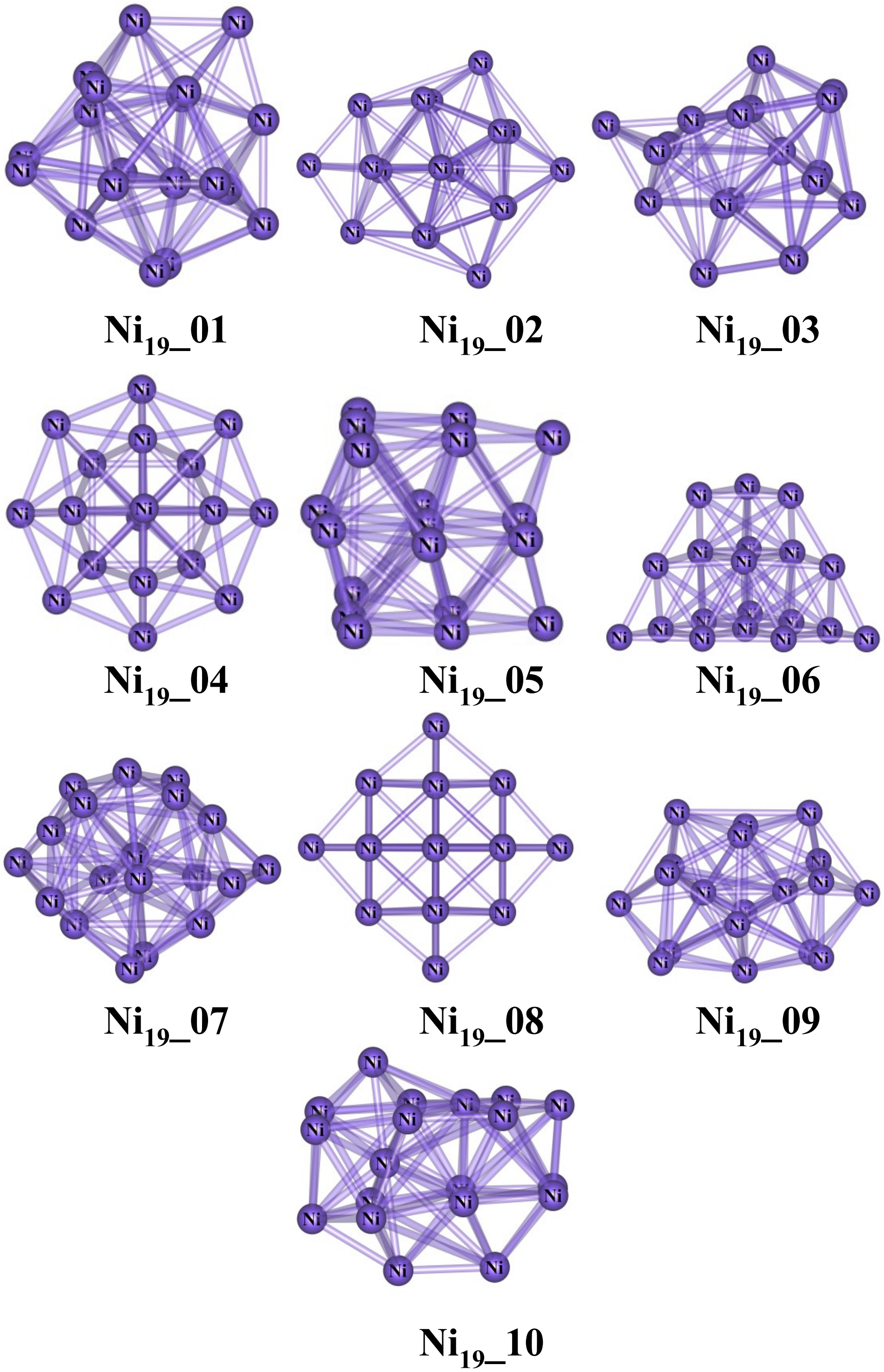

Figure S13. Illustration of nickel atomic clusters, Ni_{19}, as determined through the geometric optimization process using the DFT method at B3LYP-GD3BJ/LANl2DZ. The X variable in Ni_n_X name shows different structural isomers for a specific cluster size.

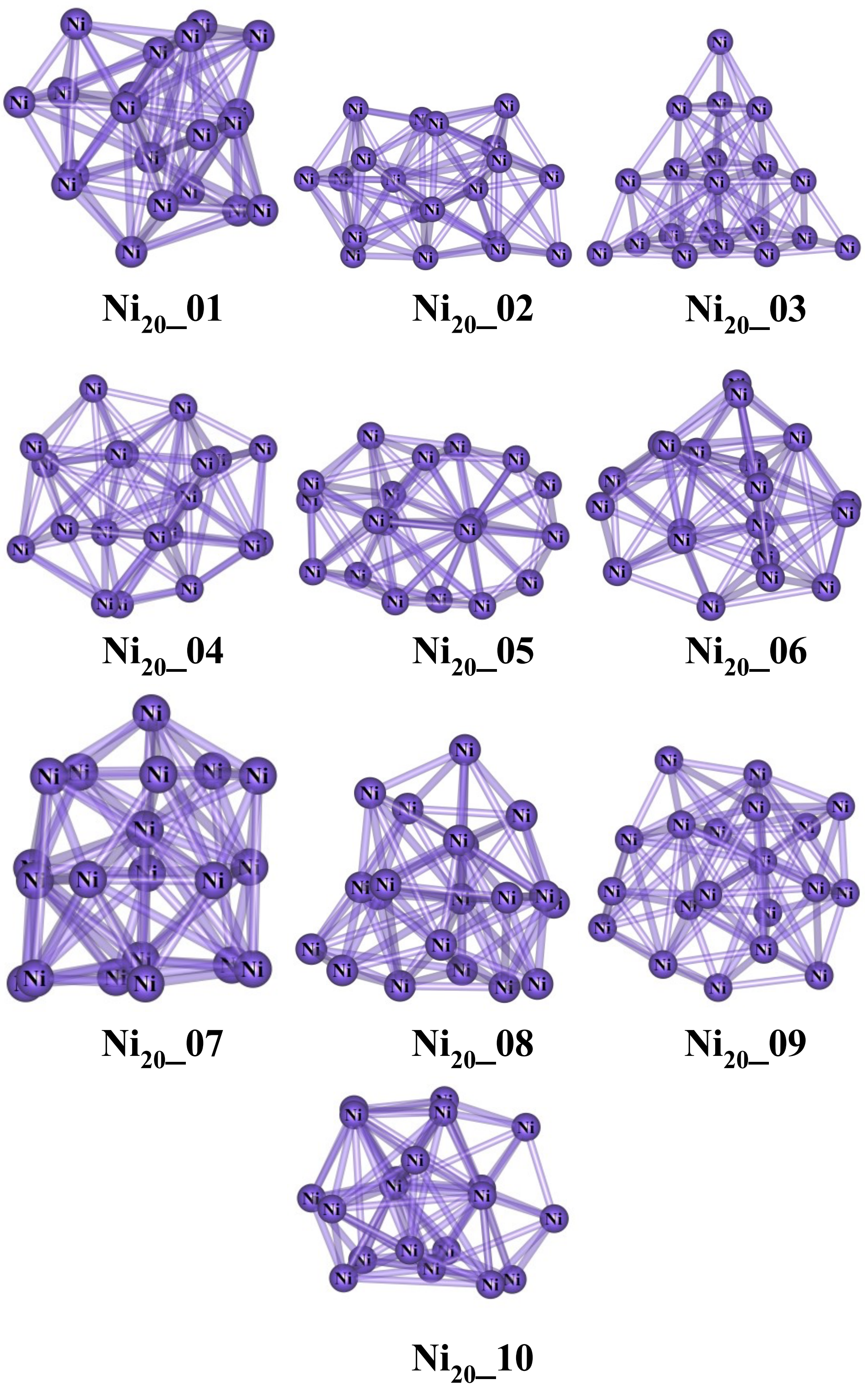

Figure S14. Illustration of nickel atomic clusters, Ni_{20}, as determined through the geometric optimization process using the DFT method at B3LYP-GD3BJ/LANl2DZ. The X variable in Ni_n_X name shows different structural isomers for a specific cluster size.

Table S7

The values of adsorption energies (E_{ads}), eV, for the most stable configurations of gas@cluster systems, including CO, CO_2, CH_4, NO, NO_2, NH_3, H_2, H_2O, N_2, O_2, and SO_2 gases and Ni_n (n = 1-20) atomic clusters. All values are calculated at the B3LYP-GD3BJ/LANl2DZ level of theory

Systems	CH_4	CO	CO_2	H_2	H_2O	N_2	NH_3	NO	NO_2	O_2	SO_2
Ni_1	-0.8248	-3.1672	-1.7999	-1.4212	-1.5371	-2.2295	-2.0504	-2.9284	-3.8216	-3.1881	-3.1346
Ni_2	-0.3533	-0.6466	-0.7846	-0.3241	-1.0973	-1.2388	-3.3357	-3.1495	-3.8666	-2.3765	-4.3741
Ni_3	-0.3245	-2.0049	-1.3502	-0.3405	-0.9662	-1.1305	-1.2628	-4.0465	-4.4875	-3.8219	-3.7161
Ni_4	-0.4413	-2.5257	-1.9774	-0.5436	-1.0192	-1.7539	-1.1005	-4.8069	-5.8588	-4.6103	-5.2647
Ni_5	-0.4518	-2.6239	-1.9392	-0.5631	-1.1143	-1.5423	-1.3478	-5.4748	-6.5831	-4.2077	-5.0711
Ni_6	-0.3862	-2.3213	-0.7975	-0.5464	-1.0496	-1.5055	-1.5037	-6.2404	-7.5469	-4.3682	-4.5238
Ni_7	-0.3040	-0.6714	-1.8308	-0.5376	-0.9571	-1.5543	-1.4451	-6.6696	-8.0531	-5.0350	-4.5561
Ni_8	-0.3325	-0.6004	-2.1480	-0.2226	-0.9474	-1.2172	-1.2944	-7.1439	-8.5899	-4.9095	-4.4911
Ni_9	0.0847	-2.1557	-1.5543	-0.3090	-0.8336	-1.4433	-1.4617	-7.9726	-8.7458	-4.9241	-5.0367
Ni_{10}	-0.3746	-2.1269	-1.7093	-0.1497	-0.5062	-1.1239	-1.4525	-8.0232	-9.7247	-4.8957	-4.5955
Ni_{11}	0.1548	-0.6376	-1.2772	-0.0171	-0.7428	-1.0707	-0.4546	-8.4526	-9.8685	-4.3004	-5.0351
Ni_{12}	-0.2812	-2.4789	-1.9660	-0.2608	-0.8549	-1.3725	-1.3720	-9.1154	-10.4025	-5.0977	-4.9884
Ni_{13}	0.1207	-0.4941	-1.9666	0.1131	-0.0631	-1.0408	-1.4545	-8.4136	-10.3157	-4.7888	-5.1764
Ni_{14}	-0.3506	-1.4181	-0.9868	0.3213	-1.0171	-0.7697	-0.6686	-8.9889	-15.3510	-4.9962	-4.9929
Ni_{15}	0.1826	-0.6395	-0.7508	-0.3247	-0.8009	-0.8178	-0.2179	-10.4148	-12.3606	-4.9767	-5.2583
Ni_{16}	-0.3000	-1.7097	-1.9762	-0.2155	-0.7265	-0.9395	-0.9898	-11.6093	-18.0072	-5.0901	-5.7747
Ni_{17}	-0.4152	-2.3567	-1.5509	-0.0980	-1.2022	-1.1455	-1.2682	-15.8097	-19.0045	-4.4218	-5.2970
Ni_{18}	0.2359	-2.0099	-1.1867	0.0744	-0.3656	-0.8083	-1.1213	-16.9561	-19.5259	-4.0357	-6.8806
Ni_{19}	-0.4939	-1.3140	-0.6107	0.9029	-0.1683	-0.3802	0.1101	-17.8105	-20.3053	-4.7212	-5.2793
Ni_{20}	-0.2276	-0.9195	-1.8958	-0.2404	-1.1573	-1.3889	-1.4236	-18.1721	-19.1353	-5.4238	-7.2063
